# Metallic Active Sites on MoO_2_(110) Surface to Catalyze Advanced Oxidation Processes for Efficient Pollutant Removal

**DOI:** 10.1016/j.isci.2020.100861

**Published:** 2020-01-23

**Authors:** Jiahui Ji, Rashed M. Aleisa, Huan Duan, Jinlong Zhang, Yadong Yin, Mingyang Xing

**Affiliations:** 1 Key Laboratory for Advanced Materials and Joint International Research Laboratory of Precision Chemistry and Molecular Engineering, Feringa Nobel Prize Scientist Joint Research Center, School of Chemistry and Molecular Engineering, East China University of Science and Technology, 130 Meilong Road, Shanghai 200237, China; 2 Department of Chemistry, University of California, Riverside, Riverside, CA 92521, USA; 3 School of Chemistry and Chemical Engineering, Southwest University, Chongqing 400715, China

**Keywords:** Inorganic Chemistry, Catalysis, Water Resources Engineering

## Abstract

Advanced oxidation processes (AOPs) based on sulfate radicals (SO_4_^⋅−^) suffer from low conversion rate of Fe(III) to Fe(II) and produce a large amount of iron sludge as waste. Herein, we show that by using MoO_2_ as a cocatalyst, the rate of Fe(III)/Fe(II) cycling in PMS system accelerated significantly, with a reaction rate constant 50 times that of PMS/Fe(II) system. Our results showed outstanding removal efficiency (96%) of L-RhB in 10 min with extremely low concentration of Fe(II) (0.036 mM), outperforming most reported SO_4_^⋅−^-based AOPs systems. Surface chemical analysis combined with density functional theory (DFT) calculation demonstrated that both Fe(III)/Fe(II) cycling and PMS activation occurred on the (110) crystal plane of MoO_2_, whereas the exposed active sites of Mo(IV) on MoO_2_ surface were responsible for accelerating PMS activation. Considering its performance, and non-toxicity, using MoO_2_ as a cocatalyst is a promising technique for large-scale practical environmental remediation.

## Highlights

• The degradation rate of PMS/Fe(II)/MoO_2_ system is 50 times higher than that without MoO_2_
• Fe(III)/Fe(II) cycle on (110) surface of MoO_2_ in PMS/Fe(II)/MoO_2_ system was confirmed
• The metal active sites exposed to MoO_2_ (110) surface are responsible for PMS activation
• Compared with MoS_2_, MoO_2_ co-catalytic system has less toxicity and no release of H_2_S

## Graphical Abstract

**Figure:**
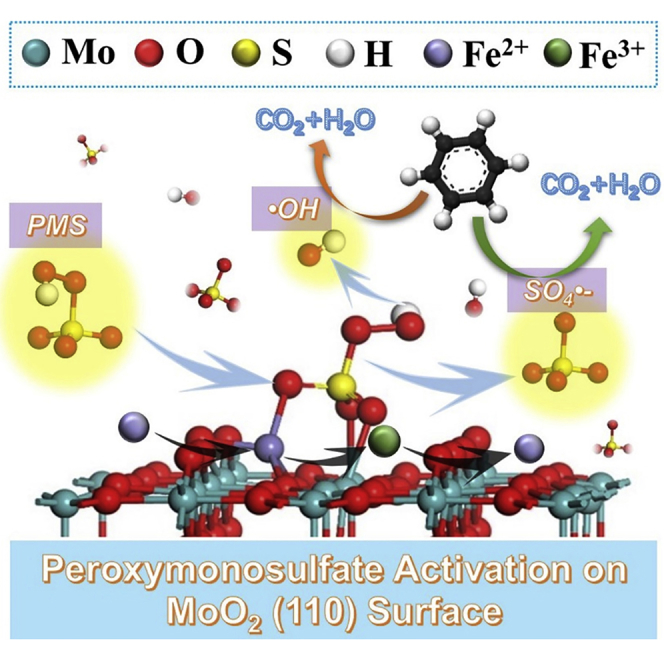

## Introduction

The presence of organic pollutants such as aromatic organic compounds in the environment is among the most significant issue for humans that requires immediate remediation (Muthuraman and Teng, 2009, Crini, 2006, Al-Ghouti et al., 2003). These pollutants are toxic, carcinogenic, and recalcitrant to degrade with time, demonstrating the great need for their removal (Ito et al., 2016, Du et al., 2018b, Yi et al., 2015, Dong et al., 2018). Although several processing methods have been proposed for effectively removing organic compounds from places such as drinking water, advanced oxidation processes (AOPs) based on the generation of hydroxyl radicals (^⋅^OH) are among the most promising techniques because they are inexpensive, environmentally safe (Buck et al., 2018, Yang et al., 2019, Tao et al., 2001), and efficient in oxidizing almost all organic pollutants to harmless products (Clarizia et al., 2017).

Recently, sulfate radical (SO_4_^⋅−^)-based AOPs have drawn much interests (Zhang et al., 2016, Yun et al., 2018, Chen et al., 2018) due to their higher oxidation potentials (SO_4_^⋅−^, 2.5–3.1 eV) compared with hydroxyl radical (^⋅^OH, 2.8 eV), longer half-life, higher selectivity (Li et al., 2018, Huang et al., 2017, Hu et al., 2017), and tolerance to wider pH range (2–8) (Ghanbari and Moradi, 2017). Peroxymonosulfate (PMS) molecules are widely used as a source for sulfate radicals in AOPs, which can be activated during the treatment process through various methods such as heating (Chen et al., 2016), UV light (Guan et al., 2011), transition metal ions, and ultrasound (Liu et al., 2017, Du et al., 2018a). Dionysiou et al. found that PMS can be activated by various transition metals, among which Co(II) and Ru(III) demonstrated the best performances as catalysts for generating sulfate radicals (Anipsitakis and Dionysiou, 2003, Anipsitakis and Dionysiou, 2004). However, their high toxicity and scarcity significantly limited their implementation in PMS activation system. A more environmental and economical alternative to Co and Ru has been found to be Fe(II), which can decompose PMS and generate SO_4_^⋅−^ in a similar manner (Dan et al., 2014). Generally, the stoichiometric ratio of PMS to Fe(II) is maintained at approximately 1:1 according to Equation 1. Transformation from Fe(III) to Fe(II) was found to be the limiting step for the reaction (Anipsitakis and Dionysiou, 2003). Besides, the activation of PMS by Fe(III) will also produce SO_5_^⋅−^ (1.1 eV) under acidic conditions (Equation 2), greatly decreasing its oxidation capacity (Anipsitakis and Dionysiou, 2004).

(Equation 1) $$Fe^{2+}+HSO_{5}^{-}\to Fe^{3+}+SO_{4}^{\cdot -}+OH^{-}$$

(Equation 2) $$Fe^{3+}+HSO_{5}^{-}\to Fe^{2+}+SO_{5}^{\cdot -}+H^{+}$$

In addition, the amount required for Fe(II) to be used in PMS activation is considered extremely large, which is also responsible for producing large amount of iron sludge (Rastogi et al., 2009b). Therefore, several other combination methods have been proposed to further enhance the performance of Fe(II) in PMS activation system. For example, iron tetracarboxyphthalocyanine molecules were synthesized as a homogeneous catalyst to activate PMS, which outperformed the performance of Co(II) (Dai et al., 2017). Also, a p-Mn/Fe_3_O_4_ catalyst with high porosity showed excellent regeneration ability just by simply washing with deionized water (Du et al., 2018a). Assisted UV irradiation has shown also to greatly improve the regeneration of Fe(II) (Khan et al., 2016). However, the following factors need to be considered when using the assisted-Fe(II)/PMS activation: (1) the elimination of competitive reaction between organic complexes and pollutants; (2) the reduction of energy consumption during the process; and (3) the simplicity of preparation and availability of the assisted materials or methods. Recently, AOPs with MoS_2_ as a cocatalyst have achieved surprising results (Xing et al., 2018, Wang et al., 2020, Sheng et al., 2019). However, there are still some critical problems with MoS_2_ as a cocatalyst to decompose PMS: the inevitable secondary pollution caused by the generation of H_2_S during reaction and the fact that MoS_2_ itself can activate PMS, leading to itself to be consumed.

Therefore, there is an urgent need to develop a greener and more efficient cocatalyst that can replace MoS_2_ for rapid, stable, and efficient cocatalytic decomposition of PMS for environmental remediation. Here, we present a facile strategy to significantly enhance the performance of SO_4_^⋅−^-based AOPs by using molybdenum dioxide (MoO_2_) as a cocatalyst in PMS/Fe(II) system. The oxidation efficiencies of PMS/Fe(II)/MoO_2_ system were examined with different aromatic organic pollutants, including lissaminerhodamine B (L-RhB), phenol, methylene blue (MB), sulfadiazine, and norfloxacin. Among them, the degradation rate of L-RhB in the PMS/Fe(II)/MoO_2_ system was significantly improved, 50 times higher than that in the PMS/Fe(II) system, with removal efficiency of 96% in 10 min while very low concentration of Fe(II) was used (0.036 mM), exceeding most reported PMS/Fe(II) systems. We further employed surface chemical analysis and DFT calculation to understand the cocatalytic enhancement of MoO_2_. The results revealed that the (110) crystal plane of MoO_2_ worked as active site for PMS activation, where the exposed Mo(IV) on the MoO_2_ cocatalyzed the conversion of Fe(III) to Fe(II). To the best of our knowledge, this is the first report of utilization MoO_2_ as a cocatalyst in SO_4_^⋅−^-based AOPs. Compared with MoS_2_ cocatalytic AOPs system, MoO_2_ cocatalytic system has higher valence band free electron density, less toxicity, better stability, and no release of secondary pollutants H_2_S (Hu et al., 2009). Therefore, it is reasonable to believe that MoO_2_ cocatalytic activation of PMS will have greater potential for large-scale practical environmental remediation.

## Results

### MoO_2_ Cocatalytic PMS/Fe(II) System for the Oxidation Reaction

**Figure 1:**
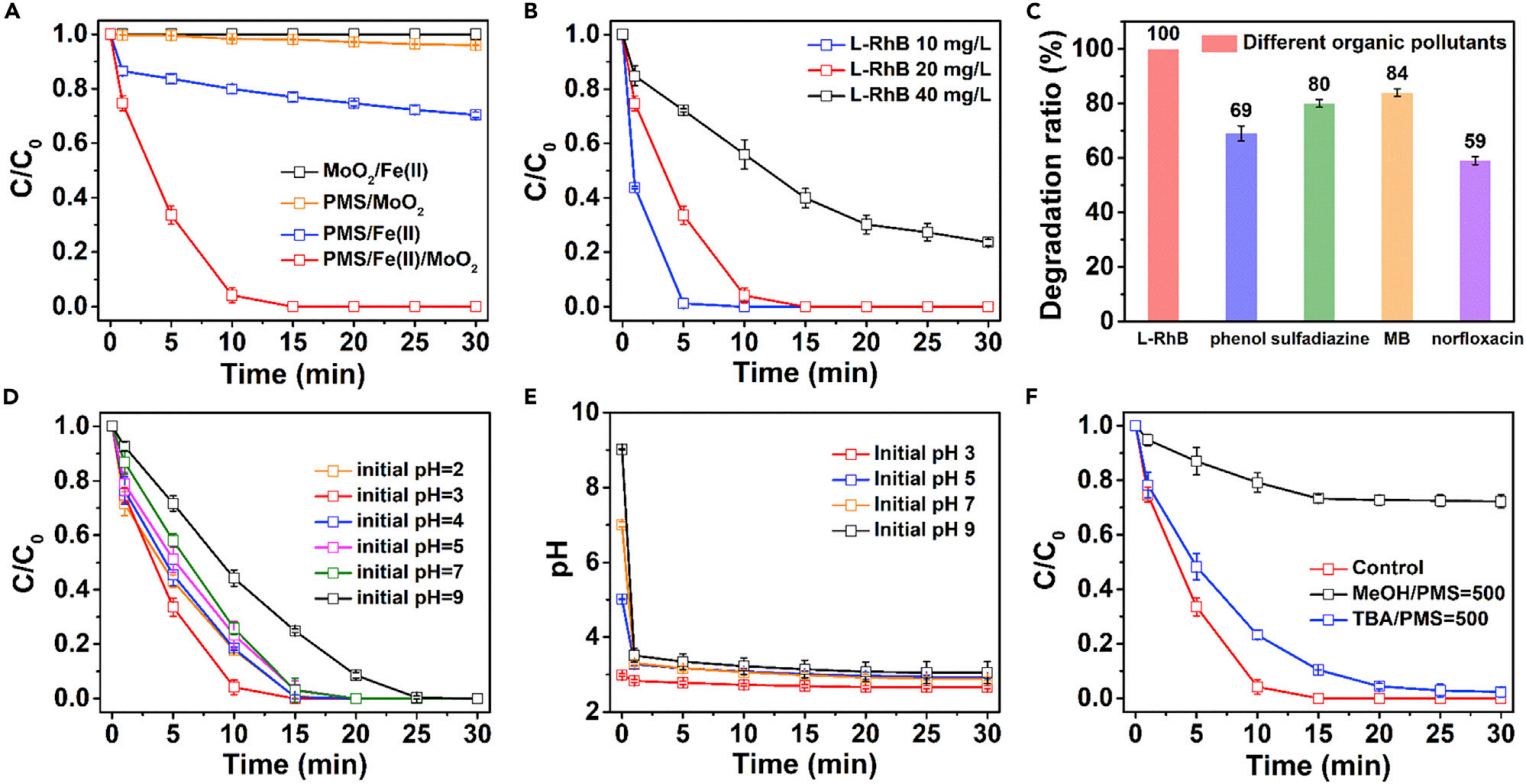
Exploration of the Best Reaction Conditions for PMS/Fe(II)/MoO_2_ System (A) Oxidation of L-RhB in different systems; oxidation of different (B) L-RhB concentrations; (C) organic compounds in the PMS/Fe(II)/MoO_2_ system; (D) the effect of initial pH and (E) variation of pH on L-RhB degradation in the PMS/Fe(II)/MoO_2_ system; (F) inhibition effect of radical scavengers on L-RhB degradation in the PMS/Fe(II)/MoO_2_ system. General conditions: [PMS]_0_ = 0.650 mM, [Fe(II)]_0_ = 0.036 mM, [MoO_2_]_0_ = 300 mg/L, initial pH = 3.0, [organic compound]_0_ = 20 mg/L. Error bars represent the standard deviation from at least duplicate experiments.

As shown in Figure 1A, no oxidation of L-RhB was observed in the absence of PMS. Besides, in the absence of Fe(II) ions, the oxidation efficiency was extremely low that only 4.1% of L-RhB was removed. This is attributed mainly to almost no production of reactive radical species in the absence of PMS or Fe(II). When the concentration of Fe(II) was fairly low (0.036 mM) and no MoO_2_ was added, the degradation performance of the PMS/Fe(II) system remained poor because of the slow conversion rate of Fe(III) to Fe(II) (Anipsitakis and Dionysiou, 2004), with only 29% of L-RhB degraded within 30 min. However, when all components were involved, L-RhB degraded near completely in 10 min (96%), indicating that MoO_2_ accelerated the conversion from Fe(III) to Fe(II), leading to continuous activation of PMS.

We also found that the degradation rate slowed as the concentration of L-RhB increased (Figure 1B), because there is always a constant number of radical species generated when the amount of PMS is fixed. In addition to L-RhB, the PMS/Fe(II)/MoO_2_ system also showed a rapid and effective degradation of other organic pollutants. Figure 1C shows that phenol, MB, sulfadiazine, and norfloxacin were degraded by 69%, 84%, 80%, and 59% in 30 min, respectively, demonstrating the potentials of this system for remediating various organic pollutants.

To explore the influence of MoO_2_, Fe(II), and PMS concentrations on the reaction rate, a series of experiments were conducted to determine the best reaction conditions (Figures S1A–S1C). The oxidation rate of L-RhB becomes faster with the increase of Fe(II) and MoO_2_ at pH 3.0 (Figures S1A and S1B). It is worth noting that the concentration of Fe(II) in the solution was extremely low (0–0.036 mM), far less than the molar amount of PMS, so the increase of Fe(II) concentration had a significant effect on the PMS activation (Anipsitakis and Dionysiou, 2003). The more addition of MoO_2_ provided more redox active sites for the transformation from Fe(III) to Fe(II), resulting in the rapid oxidation of L-RhB. However, with the increase of PMS (Figure S1C), the degradation rate first increased and then decreased a little, reaching the maximum when the adding amount of PMS was 0.650 mM, which could be attributed to the scavenging of SO_4_^⋅−^ by excess PMS to produce SO_5_^⋅−^ (1.1 eV) via Equation 3 (Ling et al., 2010).

(Equation 3) $$SO_{4}^{\cdot -}+HSO_{5}^{-}\to SO_{4}^{2-}+SO_{5}^{\cdot -}+H^{+}$$

The kinetics were investigated by using a first-order kinetic model, as in the following equation: −ln(*C*/*C*_*0*_) = *k*⋅*t*, where *C*_*0*_ and *C* represent organic matter concentrations at time *t* = 0 and t, respectively, and *k* (min^−1^) is the reaction rate constant (Figures S1D–S1F). Figures S1D–S1F show that the experiment results were fitting the first-order kinetics well. Not surprisingly, the reaction rate constant (*k*) was upgraded with the increase of Fe(II) and MoO_2_. Specifically, the *k* value with the condition of 0.036 mMFe(II) (0.311 min^−1^) was 222 times faster than that without Fe(II) (0.00140 min^−1^). Meanwhile, the addition of MoO_2_ made “*k*” 4–50 times faster than that with no MoO_2_ added (0.00938 min^−1^), and there was no striking difference between 300 mg/L and 600 mg/L MoO_2_ added. When the PMS concentration was 0.650 mM, the *k* value was the largest, about 2.3 times higher than that with 0.325 mM and a little higher than that with 1.300 mM. As a result, we concluded that Fe(II) had the greatest influence on the reaction rate in the PMS/Fe(II)/MoO_2_ system, whereas the addition of MoO_2_ significantly reduced the required amount of Fe(II), and the amount of PMS greatly determined the amount of radical species generated.

In the exploration of the influence of the initial pH in the mixture, we found that L-RhB could be removed efficiently in 30 min with a wide initial pH range of 2.0–9.0, as shown in Figure 1D. An increase in the degradation efficiency of L-RhB was obtained by increasing the initial pH from 2.0 to 3.0, in which Fe(OH)_2_ might form and activate PMS more efficiently as reported previously (Pignatello et al., 2006). However, L-RhB could be still completely oxidized within 20 min when the initial pH was neutral. There was a slight decrease in the degradation rate when the initial pH increased from 4.0 to 7.0. It has been reported that Fe(II) coprecipitates with Fe(III) oxyhydroxides when both Fe(II) and Fe(III) coexist under a pH value over 3.0. The degradation rate of L-RhB continued to decrease as the initial pH was increased to 9.0 because of more iron coprecipitation. Thus, the fastest degradation rate was obtained at pH 3.0. According to Equation 1, when Fe(II) activates PMS, OH^−^ is generated. Under acidic conditions, the generated OH^−^ can be quickly neutralized so that the reaction can proceed in the positive reaction direction. Moreover, under acidic conditions, Fe(II) is not easily complexed with OH^−^, which leads its precipitation. Thus, PMS can be activated more easily by Fe(II) under acidic conditions. Nevertheless, with the increase in initial pH, the removal efficiency of L-RhB in the PMS/Fe(II)/MoO_2_ system varied slightly but remained superior compared with the PMS/Fe(II) system. The variation of pH values in the system was also measured during the reaction process as shown in Figure 1E. Considering the possibility of radical consumption or complexation with Fe(II) or Fe(III), there were no buffering reagents included in the solution so far. Regardless of the initial pH of the system, the reaction solution would quickly become acidic when PMS was added, because KHSO_4_ molecules are essential part of the PMS mixtures (Wacławek et al., 2015). Also, the pH values slowly declined until PMS was completely consumed (Figure 1E). This explains why the PMS/Fe(II)/MoO_2_ system maintained a high level of activity in the treatment of neutral dye solution because this dropping of pH value would suppress the precipitation of Fe(II), keeping Fe(II) at high catalytic activity in the acidic solution. Moreover, the influence of solution pH was also investigated with potassium hydrogen phthalate (C_8_H_5_KO_4_, pH 4.00), mixed phosphate (pH 6.86), and borax (Na_2_B_4_O_7_⋅10H_2_O, pH 9.18) buffer solutions, respectively. As shown in Figure S2, the degradation efficiency of L-RhB became extremely poor at all three different pH conditions, which may be attributed to the consumption of most of the free radicals by the ions in the buffer solution, leading to few free radicals attacking L-RhB molecular (Zou et al., 2013).

We concluded that the optimal conditions for the degradation of L-RhB were as follows: an initial pH value of 3.0, PMS concentration of around 0.650 mM, and the more MoO_2_ and Fe(II) are added to the system, the faster the reaction rate will be. Given that moderate dosages of 300 mg/L MoO_2_ and 0.036 mM Fe(II) were enough to completely degrade L-RhB, they were chosen for most further experiments in the subsequent investigations. Ultimately, the performance of PMS/Fe(II)/MoO_2_system was also compared with other reported heterogeneous catalysis SO_4_^⋅−^-based AOPs, where its removal efficiency performed most reported values as shown in Table S1.

### Identification of Reactive Species in the PMS/Fe(II)/MoO_2_ System

KHSO_5_ has an asymmetric structure (HO-O-SO_3_^−^), so it can be activated to produce sulfate radical (SO_4_^⋅−^) via Equation 1, persulfate radical (SO_5_^⋅−^) via Equation 2, or hydroxyl radicals (^⋅^OH) via Equation 4. At the same time, those radicals interconvert via Equations 5 and 6, which is partially influenced by the solution pH (Duan et al., 2018). For further exploration of the main reactive species throughout the organic oxidation process, selective radical quenching tests were done with TBA and MeOH. The carbon atom of MeOH attached to the hydroxyl has three α-hydrogens [(α-H)_3_-C-OH), which allows methanol to capture ^⋅^OH (k = (1.2−2.8) × 10^9^ M^−1^⋅s^−1^) and SO_4_^⋅−^ (k = (1.6−7.7) × 10^7^ M^−1^⋅s^−1^)] at high reaction rates. On the other hand, TBA, which has no α-hydrogen, can react with ^⋅^OH (k = (3.8−7.6) × 10^8^ M^−1^⋅s^−1^) faster than SO_4_^⋅−^ (k = (4.0−9.1) × 10^5^ M^−1^⋅s^−1^) (Liang and Su, 2009). However, both MeOH and TBA are nonreactive with SO_5_^⋅−^ (k ≤ 10^3^ M^−1^⋅s^−1^) (Hayon et al., 1972). Therefore, the contributions of SO_5_^⋅−^ and ^⋅^OH/SO_4_^⋅−^ can be differentiated by MeOH, whereas TBA can be employed to distinguish the contributions of ^⋅^OH and SO_4_^⋅−^.

(Equation 4) $$Fe^{2+}+HSO_{5}^{-}\to Fe^{3+}+SO_{4}^{2-}+\cdot OH$$

(Equation 5) $$SO_{4}^{\cdot -}+OH^{-}\to SO_{4}^{2-}+\cdot OH$$

(Equation 6) $$HSO_{4}^{-}+\cdot OH\to SO_{4}^{\cdot -}+H_{2}O$$

**Figure 2:**
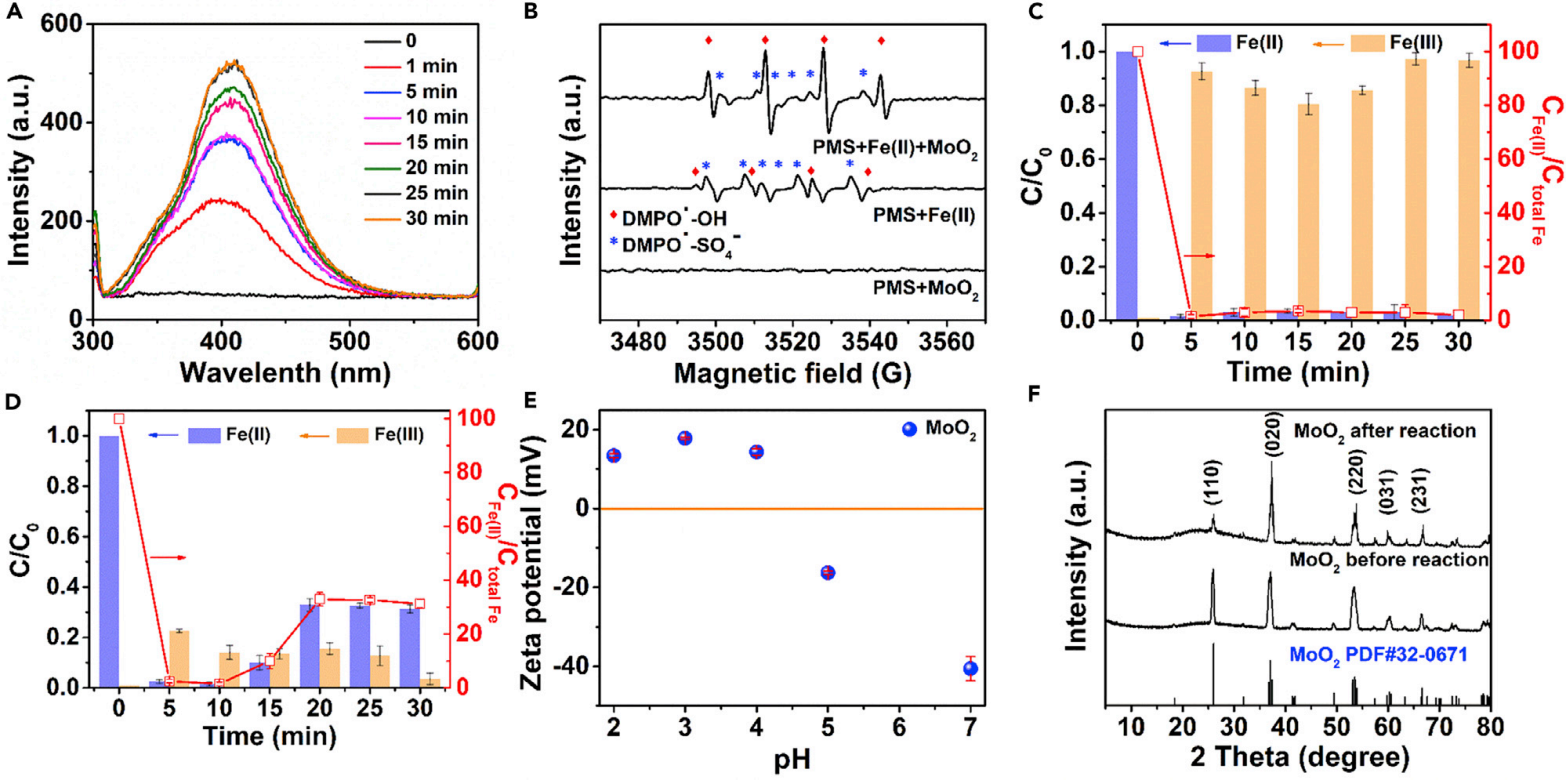
Exploration of Reactive Species and Reaction Mechanism (A) Photoluminescence spectra of benzoic acid mixed with the PMS/Fe(II)/MoO_2_ system; (B) EPR spectra obtained from the PMS/MoO_2_ system, PMS/Fe(II) system, and PMS/Fe(II)/MoO_2_ system with the existence of DMPO (◆ represents DMPO^⋅^-OH adduct and * represents DMPO^⋅^-SO_4_^−^ adduct); the variation of Fe(II) and Fe(III) concentrations in (C) the PMS/Fe(II) system; (D) the PMS/Fe(II)/MoO_2_ system; (E) zeta potential of MoO_2_ at different pH values; (F) XRD patterns of MoO_2_ before and after the reaction. General conditions: [PMS]_0_ = 0.650 mM, [Fe(II)]_0_ = 0.036 mM (total Fe), [MoO_2_]_0_ = 300 mg/L, initial pH = 3.0, [L-RhB]_0_ = 20 mg/L. Error bars represent the standard deviation from at least duplicate experiments.

As shown in Figure 1F, when the molar ratio of MeOH to PMS was maintained as 500:1, only 26% of L-RhB was degraded, which confirms the small contribution of SO_5_^⋅−^ in the system. However, 100% degradation efficiency was achieved in 30 min when 500 times molar ratio of TBA to PMS was maintained in the mixture, which was much slower compared with the controlled experiment. This result indicates that the radicals generated from PMS were mainly SO_4_^⋅−^, ^⋅^OH, and a small number of SO_5_^⋅−^. The presence of Fe(II) under acidic conditions implies that SO_4_^⋅−^ and ^⋅^OH contributed the most to L-RhB degradation. To further prove the generation of ^⋅^OH, the photoluminescence (PL) signal of hydroxybenzoic acid formed by benzoic acid capturing ^⋅^OH was measured. As shown in Figure 2A, the signal of hydroxybenzoic acid increased significantly in the first five minutes and then slowed down, which is consistent with the interpretation that ^⋅^OH plays a significant role in the system.

To further support these assumptions, electron paramagnetic resonance (EPR) was employed to detect the existence of radicals, coupled with 5,5-dimethyl-1-pyrroline (DMPO) as a spin-trapping reagent that can capture both SO_4_^⋅−^ and ^⋅^OH. The intensity of DMPO radical adducts is in direct proportion to the concentration of reactive radical species (Zamora and Villamena, 2012, Fang et al., 2017). As illustrated in Figure 2B, compared with the low EPR signals in the PMS/Fe(II) system and no EPR signal in the PMS/MoO_2_ system, the PMS/Fe(II)/MoO_2_ system exhibited the characteristic DMPO^⋅^-OH and DMPO^⋅^-SO_4_^−^ adduct signals, which further indicates that both ^⋅^OH and SO_4_^⋅−^ were generated during PMS activation. The addition of MoO_2_ only facilitated the recycle of Fe(III)/Fe(II), hence promoting the generation of radical species. Moreover, the intensity of DMPO^⋅^-SO_4_^−^ adduct signals was much lower than the DMPO^⋅^-OH adduct signals. This might be attributed to the fast conversion of DMPO^⋅^-SO_4_^−^ adducts to DMPO^⋅^-OH adducts through the nucleophilic substitution reaction (Furman et al., 2010, Timmins et al., 1999).

### Exploration of PMS Activation Mechanism in PMS/Fe(II)/MoO_2_ System

The slow conversion of Fe(III) to Fe(II) is the rate-determining step in effective PMS activation (Rastogi et al., 2009a, Rastogi et al., 2009b). Based on our results, the acceleration of L-RhB oxidation rate was attributed to MoO_2_ promoting the transformation of Fe(III) to Fe(II), consequently leading to faster activation of PMS. To further explore this hypothesis, the valence levels of Fe(II) and Fe(III) during the reaction were investigated. According to Equation 4, the ratio of Fe(II) to Fe(III) is believed to be positively correlated with the activation rate of PMS. 1,10-phenanthroline molecule can complex with Fe(II) to produce the jacinth complex in a pH range of 2–9 (Harvey et al., 1955, Herrera et al., 1989), whereas potassium thiocyanate (KSCN) is usually used to coordinate with Fe(III) to produce a blood-red complex (Kusic et al., 2011). As shown in Figures 2C and 2D, before the addition of PMS, the concentrations of Fe(II) (blue bar) were the same, whereas no Fe(III) was detected in the solutions (orange bar) in both the PMS/Fe(II) system and the PMS/Fe(II)/MoO_2_ system. When PMS was added, the concentrations of Fe(II) in the solutions rapidly decreased, and the concentrations of Fe(III) reached their maximum values within 5 min, illustrating that most Fe(II) was immediately oxidized to Fe(III) by PMS (Equation 1), and the reduction of Fe(III) was slow in the system (Equation 2). Fe(II) was extremely low during L-RhB oxidation in both systems. After almost complete consumption of PMS, Fe(III) was gradually reduced to Fe(II) by MoO_2_ until it maintained a relative dynamic equilibrium with the residual PMS, further indicating that MoO_2_ continuously accelerate the conversion of Fe(III) to Fe(II) because the presence of PMS made Fe(II) difficult to exist stably. After the PMS was almost consumed, the stable existence of Fe(II) could be detected. Notably, the equilibrium concentration of Fe(III) in the PMS/Fe(II)/MoO_2_ system was much lower than that in the PMS/Fe(II) system. Therefore, zeta potential tests were conducted to determine the isoelectric point (IEP) of MoO_2_. The results showed that its IEP was between pH 4 and 5 (Figure 2E). Because the pH was lower than 4 during the reaction, the surface of MoO_2_ would be positively charged, leading PMS to be easily adsorbed, and then Fe(II) could be absorbed as well. Then, MoO_2_ was recovered, dried, and redispersed in an acidic aqueous solution (pH = 3) after completing the oxidation reaction. Through ICP measurements of the supernatant, we found that the iron ions adsorbed on the surface of MoO_2_ accounted for 87.7% of the initial amount, which could explain the low equilibrium concentration of Fe(III) and the incomplete recovery of Fe(II) in the PMS/Fe(II)/MoO_2_ system.

(Equation 7) $$2Fe^{3+}+\equiv Mo^{4+}\to 2Fe^{2+}+\equiv Mo^{6+}$$

(Equation 8) $$Fe^{3+}+\equiv Mo^{4+}\to Fe^{2+}+\equiv Mo^{5+}$$

**Figure 3:**
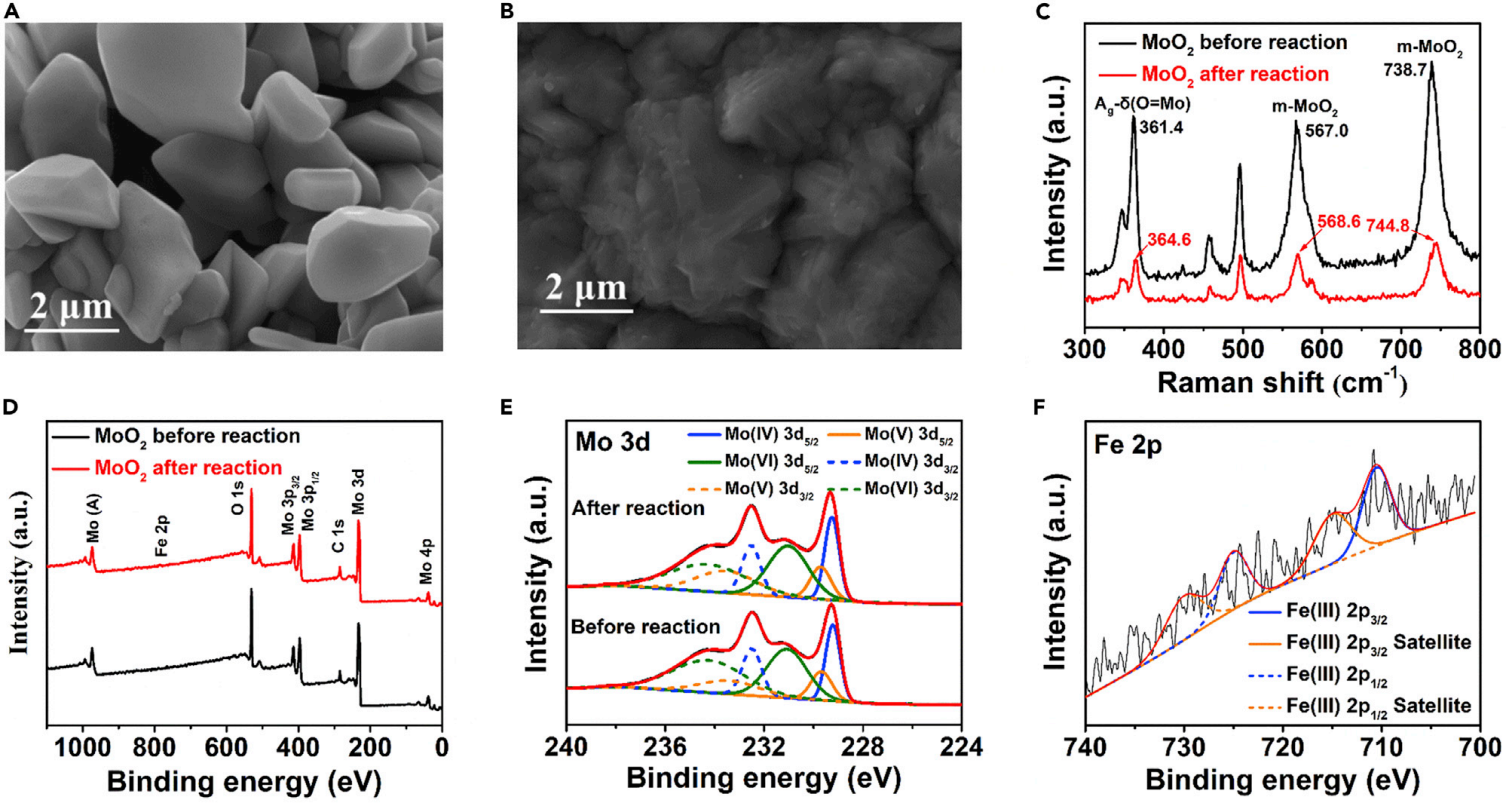
Characterization of MoO_2_ before and after the Reaction SEM images of MoO_2_ (A) before and (B) after reaction; (C) Raman spectra of MoO_2_ before and after reaction; (D) XPS survey spectra and (E) Mo3d spectra of MoO_2_ before and after reaction; (F) Fe2p spectra of adsorbed iron after reaction in PMS/Fe(II)/MoO_2_ system.

Given that the reduction potential of Fe(III)/Fe(II) (0.77 V) is higher than that of MoO_4_^2−^/MoO_2_ (0.65 V) (Du et al., 2018a), it could be speculated that Mo(IV) on the surface of MoO_2_ was oxidized by Fe(III) to Mo(V) and Mo(VI) (Equation 7). Fe(III) was converted to Fe(II) simultaneously (Equation 8), which was supported by Figure S3. (Ugoet al., 2002) To further support this argument, we studied the surface conditions of MoO_2_ via SEM, XRD, Raman, and XPS, as depicted in Figures 2F and 3. Figures 3A and 3B display the SEM images of MoO_2_ before and after reaction. It can be seen that the surface of MoO_2_ after reaction was much rougher than that before the reaction, which proves that MoO_2_ participated in the reaction. However, the XRD spectra in Figure 2F shows that the crystalline structure of MoO_2_ did not change after the reaction, demonstrating that the deformed monoclinic structure of MoO_2_ was quite stable, but the relative strength of the crystal plane (110) decreased, which might be ascribed to the redox reaction taking place on this plane and changing its surface condition (Xie et al., 2015, Sun et al., 2011). Moreover, the surface property of MoO_2_ was investigated by Raman spectroscopy. The variety of electron cloud density causes red/blue shift of Raman peaks. As shown in Figure 3C, A_g_-δ(O=Mo) peak and two m-MoO_2_ peaks of MoO_2_ are blue shifted by 3, 1, and 6 cm^−1^, respectively, after reaction (Camacho-López et al., 2011), because the electron clouds on the surface of MoO_2_ transfer to Fe(III), leading to the decrease of the probability of collision between photons and electrons, so that the average free path of collision increases and the energy loss caused by collision decreases. Therefore, the energy of photons scattered by MoO_2_ after reaction is higher than that of the ones scattered by MoO_2_ before reaction, causing the displacement of three peaks of Raman spectra, the oxidation of Mo(IV), and the reduction of Fe(III). The variety of valence state of Mo in MoO_2_ was evaluated by X-ray photoelectron spectroscopy (XPS). Five distinct peaks in the survey spectra of the MoO_2_ before and after reaction are exhibited in Figure 3D, which can be indexed to Mo 3d (232.7 eV), C 1s (284.7 eV), Mo 3p (396.7 and 413.7 eV), and O 1s (530.7 eV), respectively. The Mo 3d peaks were further explored by high-resolution XPS. Figure 3E shows the multiple peak of Mo 3d spectra, which are fitted well into three spin-orbit doublets, coinciding to the peaks of Mo(IV), Mo(V), and Mo(VI) oxidation states. In detail, the two Mo 3d peaks of MoO_2_ before/after reaction centered at 229.2/229.3 and 232.5/232.5 eV can be attributed to Mo(IV) 3d_5/2_ and Mo(IV) 3d_3/2_, the two peaks located at 229.7/229.7 and 233.4/233.5 eV are indexed to Mo(V) 3d_5/2_ and Mo(V) 3d_3/2_ (Zhang et al., 2019, Barros et al., 2003, Yi et al., 2019), and the other two peaks located at 231.1/231.0 and 234.3/234.3 eV are inferred to Mo(VI) 3d_5/2_ and Mo(VI) 3d_3/2_ (Camacho-López et al., 2011, Hanawa et al., 2001, Xie et al., 2015). Detailed fitting data are listed in Table S2 and the peak area ratios of Mo(IV)/(Mo(V)+Mo(VI)) are calculated, which varies from 0.355 to 0.346, manifesting that some of Mo(IV) on the sample surface was oxidized to Mo(V) and Mo(VI), leading to a slight decrease of the ratios. Fe ions (0.21 at.%) were also detected on the surface of MoO_2_, which is consistent with the result of the ICP test, but it is difficult to split the peak of Fe2p high-resolution XPS due to the low content of Fe. As shown in Figure 3F, Fe(III) and its satellite peaks are fitted (Tang et al., 2015), proving the existence of Fe(III) on the surface of MoO_2_. Moreover, as shown in Figure S4, almost no change was found between O1s spectra of MoO_2_ before and after reaction (Xia et al., 2018), indicating that no iron oxide was formed.

### DFT Calculation

DFT calculation was employed to investigate the reaction mechanism in the PMS/Fe(II)/MoO_2_ system. MoO_2_ has a monoclinic crystal structure, with P21c space group, and unit cell dimensions of a = 5.611 Å, b = 4.856 Å, c = 5.629 Å, and β = 120.95° (Brandt, 1971). Figure S5A shows its crystal structure, which consists of distorted octahedral [MoO_6_] units. Structural optimizations of bulk MoO_2_ were performed at a series of volumes to obtain the equilibrium unit cell parameters. The calculated lattice parameters (a = 5.594 Å, b = 4.910 Å, c = 5.682 Å) and bond angle (β = 120.47°) were generally consistent with experimental data. To better understand the activation mechanism of PMS molecules (labeled as HSO_5_^−^ in Figure S5B) on the MoO_2_ surfaces, DFT calculations were performed to determine which species are stable. The most commonly studied surface in rutile-type MoO_2_ systems is the (110) plane, where the atomic layers along the [110] direction are ordered as MoO-O-O′-MoO (Tokarz-Sobieraj et al., 2011). The MoO_2_ (110) surface possesses three distinct surface terminations: (1) both Mo and O atoms exposed, (2) with O atoms exposed, and (3) O′ atoms exposed, as shown in Figures S5C–S5E. The comparison of surface formation energy—1.25 J/m^2^, 1.12 J/m^2^, and 0.79 J/m^2^—indicated that a surface with the “bridging oxygen” termination (O′ termination) was most likely to form, hence, it was selected for the further analysis.

**Figure 4:**
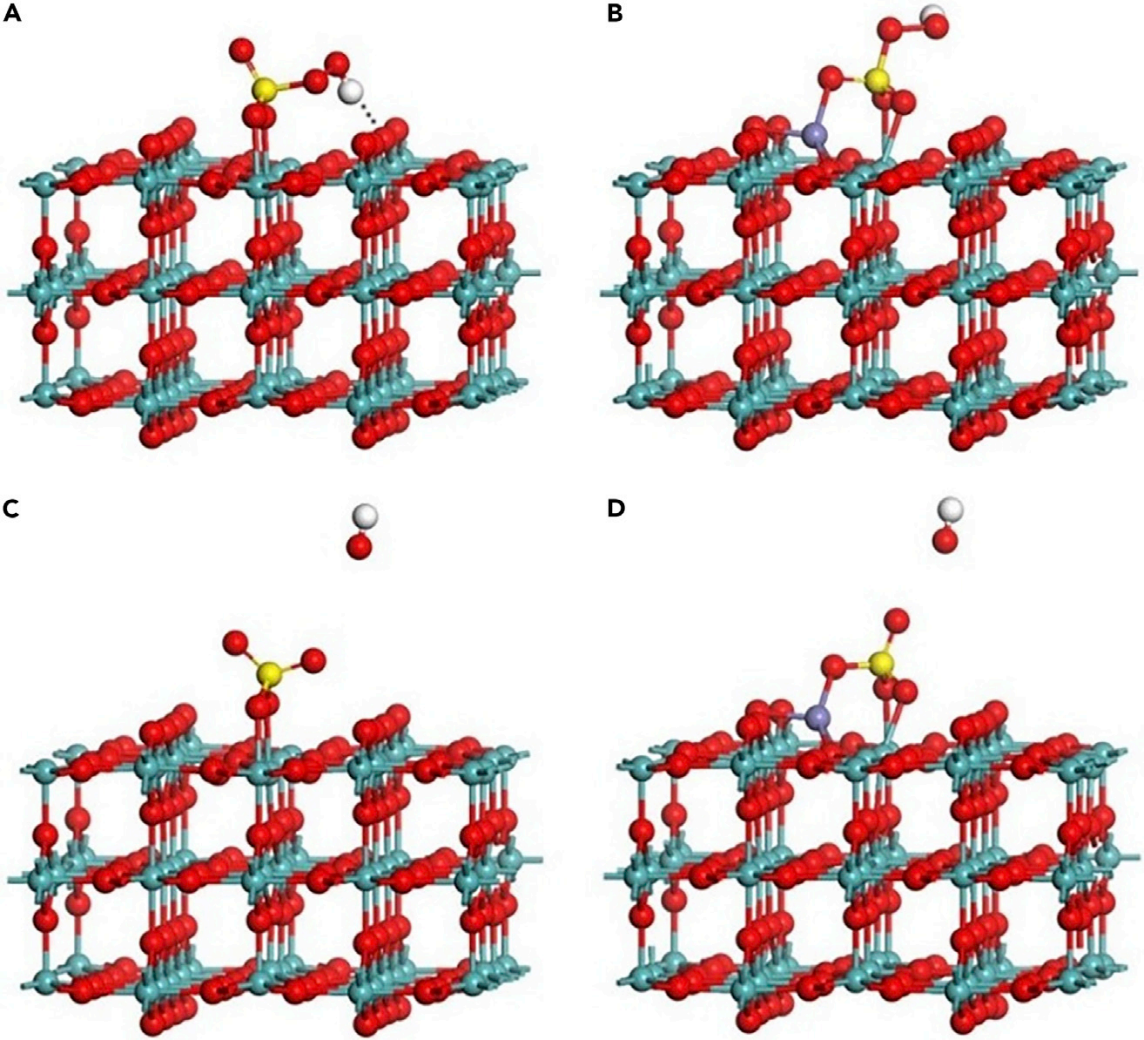
DFT Calculation of PMS Activation on MoO_2_ (110) Surface The optimal adsorption configuration of PMS and its decomposition on MoO_2_ (110) surfaces, respectively. Only side views are presented here: (A) HSO_5_^−^ on the (110) surface, (B) HSO_5_^−^ on the Fe(II)-decorated (110) surface, (C) SO_4_^2−^+HO˙ on the (110) surface, and (D) SO_4_^2−^ + HO˙ on the Fe(II)-decorated (110) surface. The yellow, red, olive, purple, and white atoms are S, O, Mo, Fe, and H atoms, respectively.

As shown in Figure 4A, during the activation on the MoO_2_ (110) surface, the PMS molecule was likely to locate at the MoO_2_ (110) surface with the two O atoms on the -SO_4_ side bonding with two Mo atoms of the surface. The two bond lengths were calculated as 2.09 Å and 2.07 Å, respectively. In addition, the H atom on the -OH side would form a hydrogen bond with the O′ termination (approximately 1.80 Å in length), where the O-O bond length (*l*_O-O_) rarely changed after its adsorption. All these inhibited the generation of hydroxyl radicals, which could explain the poor performance of MoO_2_ alone in activating PMS. For the adsorption of PMS on the Fe(II)-decorated O′ surface, the PMS attached to the surface with three O atoms from -SO_4_ group binding the Fe(II) and two Mo atoms, as shown in Figure 4B. The bond lengths were calculated as 2.08 Å, 2.24 Å, and 2.27 Å, respectively. The adsorption between PMS and surface was enhanced by these three bonds, the occurrence of more electron transfer, and that -OH side would be maintained far from the surface, leading to an elongation of *l*_O-O_. To better understand the interaction between the surfaces and PMS activation, we calculated the adsorption energy of PMS (*E*_ads_) on the different surfaces, charge transfer (Δq) between PMS and (110) surfaces, and the bond length (*l*_O-O_) between the -OH group and -SO_4_ group. All results are summarized in Table S3. The adsorption on both surfaces was found to be strong, with *E*_ads_ being −2.06 and −3.17 eV for MoO_2_ (110) surface without and with Fe(II) respectively. This was also consistent with the formation of chemical bonds between PMS and the two surfaces, illustrating the strong interaction between PMS and Fe(II) and electrons transferred from the surface atoms to the PMS molecules. The adsorption of PMS on Fe(II)-(110) was stronger, with lower *E*_ads_, longer *l*_O-O_, and more electrons received from the metal atoms on the surface. Therefore, we concluded that the PMS on the modified MoO_2_ (110) surface was the most active site.

**Figure 5:**
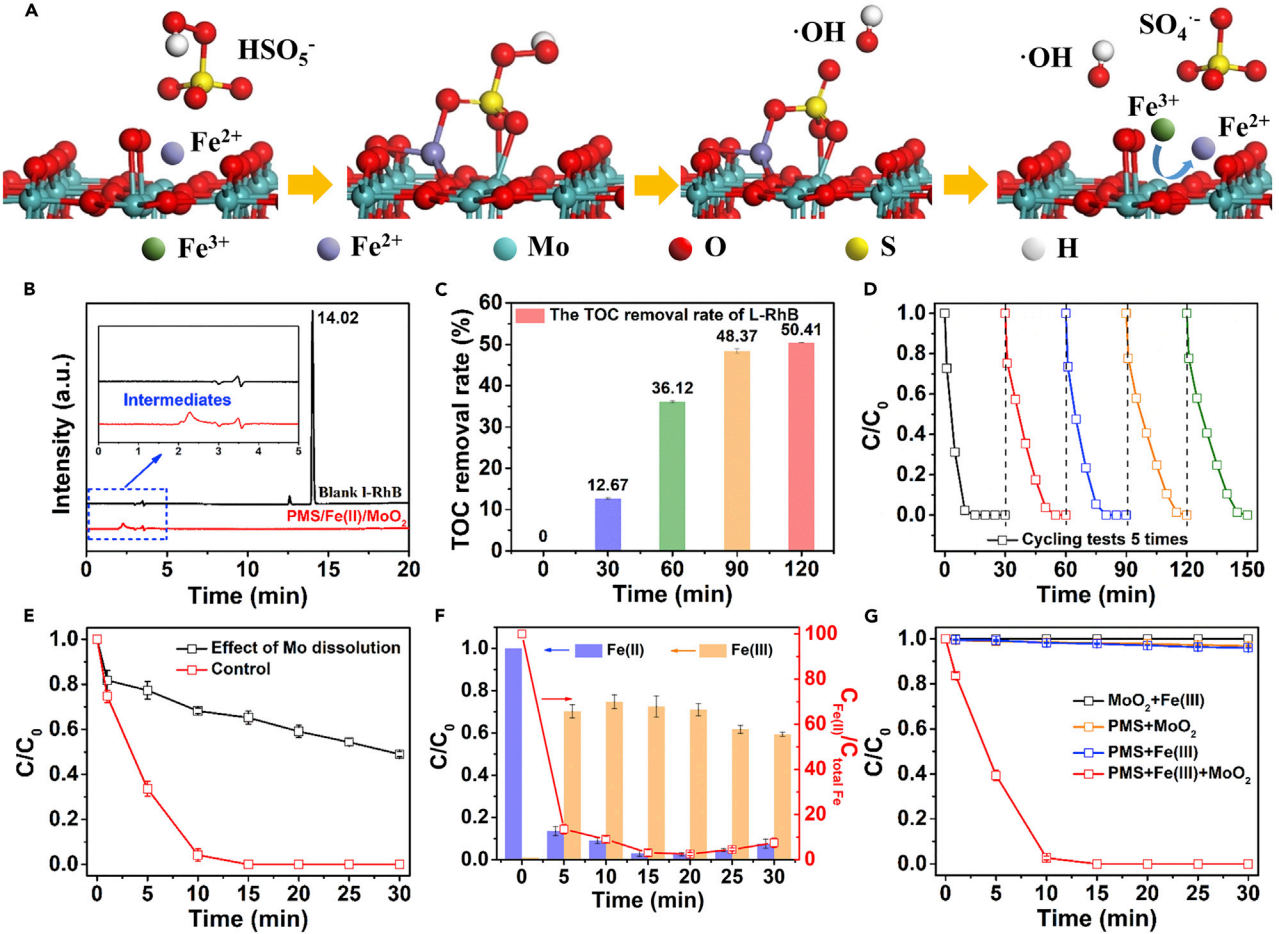
Mineralization Ability of PMS/Fe(II)/MoO_2_ System and Cyclic Stability of MoO_2_ (A) Mechanism of MoO_2_ accelerating Fe(III)/Fe(II) cycle and promoting PMS activation; (B) HPLC signals of L-RhB and intermediates; (C) TOC removal rate with 0.650 mM PMS added per 30 min; (D) cycling test of MoO_2_ (after UV irradiation); (E) effect of dissolved Mo ions on the degradation of L-RhB in PMS/Fe(II)/MoO_2_ system; (F) the variation in Fe(II) and Fe(III) concentration in PMS/Fe(II)/dissolved Mo system; (G) degradation of different L-RhB concentration in PMS/Fe(III)/MoO_2_ system. General conditions: [PMS]_0_ = 0.650 mM, [Fe(II)]_0_ = 0.036 mM (total Fe) or [Fe(III)]_0_ = 0.035 mM, [MoO_2_]_0_ = 300 mg/L, initial pH = 3.0, [L-RhB]_0_ = 20 mg/L. Error bars represent the standard deviation from at least duplicate experiments.

Based on the above comprehensive characterization and DFT calculations (Figure 4), the mechanism of the L-RhB degradation can be inferred as follows: first, HSO_5_^−^ adsorbed on MoO_2_ surface under acidic conditions, followed by Fe(II) approaching the surface owing to its positive charge. Subsequently, Fe(II) donates one electron to HSO_5_^−^ transforming into Fe(III). Therefore, HSO_5_^−^ is dissociated into the radical species (^⋅^OH and SO_4_^⋅−^) to attack the organic molecules. These results are supported by the rapid decline of Fe(II) in the first minute (Figure 2D) and the EPR signals of DMPO^⋅^-OH and DMPO^⋅^-SO_4_^−^ adducts (Figure 2B). Afterward, the organic compounds are mineralized by those radical species, and Fe(III) is reduced to Fe(II) by Mo(IV) on the surface of the MoO_2_ to continue activating PMS at the same time. Moreover, PMS is also decomposed to produce SO_5_^⋅−^ as a by-product. This cocatalytic mechanism of MoO_2_ in the PMS/Fe(II)/MoO_2_ system is schematically summarized in Figure 5A.

High-performance liquid chromatography (HPLC) was employed to analyze the primary products after the L-RhB degradation in the PMS/Fe(II)/MoO_2_ system. As shown in Figure 5B, the strongest peak at 14.02 min, which corresponds to complete disappearance of L-RhB molecules after the oxidation reaction, confirms its complete degradation. Moreover, the PMS/Fe(II)/MoO_2_ system achieved relatively a high total organic carbon (TOC) removal rate (50%) with the addition of 0.650 mM PMS per 30 min, as illustrated in Figure 5C. This method may be an appropriate way for further mineralization of intermediates to H_2_O and CO_2_ (Zou et al., 2013).

Due to the complex structure of L-RhB, we explored the degradation intermediates and mechanisms of phenol, another organic pollutant that can be degraded in the PMS/Fe(II)/MoO_2_ system. Based on the fragment peaks obtained from gas chromatography-mass spectrometry (GC-MS) measurements (Figure S6), we speculated that mainly SO_4_^⋅−^ and ⋅OH would attack the benzene ring first to form phenoxy radicals, thereby producing a series of ring-opening reactions, as speculative in the oxidation reaction pathway depicted in Scheme S1. However, the fragment (m/z = 73) with the strongest molecular ion peak could be attributed to glyoxylic acid intermediate, which is known to resist mineralization (Pimentel et al., 2008).

The reusability of MoO_2_ is a very important aspect for commercial pollutants treatment. The cocatalytic activity of MoO_2_ was greatly reduced in the second cycle as shown in Figure 5D. Vacuum calcination was employed to restore the activity of MoO_2_. As shown in Figure S7, the activity of MoO_2_ after vacuum calcination was still much worse than the original. Therefore, we suspect that the active sites on the surface of MoO_2_ were covered by carbon deposits, which were difficult to remove, but after UV irradiation of MoO_2_, its cocatalytic activity was restored, which could be attributed to the decomposition of some unmineralized carbon-based residues on MoO_2_ surface. Hence, its cocatalytic activity remained stable for the next three recycles.

Subsequently, the amount of the dissolved Mo ions under acidic conditions was determined. Figure S8 shows that the dissolution balance of Mo ions (1.60 mg/L, 0.71% of the total Mo addition) was achieved in 120 min. Because each experiment ended in 30 min, and the dissolved Mo ions might be the primary cocatalyst in reducing Fe(III) rather than MoO_2_ itself, the degradation of L-RhB and the variation of Fe(II) and Fe(III) concentrations were measured in the PMS/Fe(II)/dissolved Mo ion system. As shown in Figure 5E, the degradation rate of L-RhB dropped sharply, with only 51.9% degraded in 30 min, which is far slower than that in the PMS/Fe(II)/MoO_2_ system. This demonstrates that the main cocatalytic effect in the PMS/Fe(II)/MoO_2_ system comes from Mo(IV) on the surface of MoO_2_ rather than the dissolved Mo ions. Also, the variations of Fe(II) and Fe(III) concentrations can explain the poor performance of the PMS/Fe(II)/dissolved Mo ion system. As shown in Figure 5F, almost no Fe(II) was recovered after 30 min, whereas Fe(III) concentration remained almost constant similar to the PMS/Fe(II) system, which could be correlated to the low conversion rate of Fe(III)/Fe(II), confirming that the few dissolved Mo ions were not sufficient to promote rapid Fe(III)/Fe(II) conversion.

Ultimately, a large scale-up test with 1 L system was employed to examine the practicality in scaling-up the PMS/Fe(II)/MoO_2_ system for practical environmental remediations. As shown in Figure S9, PMS/Fe(II)/MoO_2_ system maintained its excellent catalytic performance compared with the PMS/Fe(II) system even in this large volume, consistent with results in Figure 1A. Moreover, we found that 12 times the amount of Fe(II) (40 mg/L per 10 min added) was required to make the degradation effect of PMS/Fe(II) system almost same as that of PMS/Fe(II)/MoO_2_ system. Therefore, the addition of MoO_2_ reduced the amount of Fe(II) needed by more than 92% and subsequently reduced the generation of iron sludge and the cost of secondary pollution treatment. Taking one ton of this wastewater as an example, the consumption of PMS and Fe(II) in MS/Fe(II) system was 0.82 $ and 0.17 $, respectively. And the consumption of PMS and Fe(II) in PMS/Fe(II)/MoO_2_ system was 0.82 $ and 0.01 $. Considering that the amount of PMS added to the two systems is the same, the cost difference between the two systems is mainly due to the amount of iron added. Therefore, the addition of cocatalyst can save 94% of the cost. This shows the great potentials of the PMS/Fe(II)/MoO_2_ system for industrial applications.

### Expanded Application of MoO_2_ in PMS/Fe(III) System

In general, Fe(III) does not readily activate PMS according to Equation 2. However, because the addition of MoO_2_ significantly promotes the conversion of Fe(III) to Fe(II), it should enhance the decomposition of PMS in PMS/Fe(III) system. To examine this hypothesis, we carried a series of testing for the degradation of L-RhB in PMS/Fe(III)/MoO_2_ system as shown in Figure 5G. The obtained results were far better than the PMS/Fe(III) system (4.1%) and the PMS/MoO_2_ system (3.3%), where no degradation was observed in the Fe(III)/MoO_2_ system. This might be because Fe(III) was reduced to Fe(II) immediately after the addition of MoO_2_, leading to its spontaneously precipitation. Therefore, the performance of the degradation of L-RhB is substantially the same as that in the PMS/MoO_2_/Fe(II) system. Figures S10 and S11 show the great degradation performance of L-RhB and other organics, and Figure S12 shows the almost same kinetic results as PMS/MoO_2_/Fe(II) system. The degradation of L-RhB in different pH was also investigated as shown in Figure S13. Radical quenching tests proved that SO_4_^⋅−^ was the main reactive species (Figure S14), which was further supported by EPR spectra (Figure S16). Typically, as shown in Figure S15, as the reaction progressed, Fe(III) rapidly decreased and Fe(II) gradually increased, but the total amount of iron ions detected after starting the reaction was lower than initially added. This may be because in the presence of PMS and MoO_2_, Fe(II) was rapidly oxidized by PMS, and Fe(III) was also rapidly reduced by MoO_2_, so that 1,10-phenanthroline and KSCN were difficult to capture Fe(II) or Fe(III) quickly. The result proves the circulation of iron ions during the reaction in PMS/Fe(III)/MoO_2_ system. The oxidation mechanism of L-RhB in the PMS/Fe(III)/MoO_2_ system is also basically the same as that of PMS/Fe(II)/MoO_2_ system, which was supported by SEM images (Figure S17), XRD patterns (Figure S18), Raman spectra (Figure S19), and XPS spectra (Figures S20–S22). The only difference that might exist is that in the PMS/Fe(III)/MoO_2_ system, MoO_2_ reduces the surface-adsorbed Fe(III) to Fe(II) first and then activates PMS.

## Discussion

The slow transformation from Fe(III) to Fe(II) has persistently limited the practical application of PMS/Fe(II) systems, for which a great amount of iron ions are needed to activate PMS, causing massive formation of iron sludge. In the PMS/Fe(II)/MoO_2_ system, this problem is solved by the addition of MoO_2_, which is earth-abundant, quite stable, and has enough reductive power to reduce Fe(III). Therefore, an extremely low concentration of Fe(II) (0.036 mM) is adequate to activate PMS and degrade organic pollutants rapidly in the wide pH range of 2.0–9.0. The iron sludge is limited so that no more secondary pollution is caused. SO_4_^⋅−^ and ^⋅^OH are the primary reactive species produced in the PMS/Fe(II)/MoO_2_ system. The TOC removal rate of L-RhB reached 50% with the addition of PMS, which will be an appropriate approach to completely mineralize refractory organic contaminants. Moreover, MoO_2_ could be recycled and exhibited excellent recover activity after its treatment with UV light irradiation. The involvement of MoO_2_ in the PMS/Fe(II) system could allow for the low-cost remediation of organic pollutants, thus contributing to sustainable development for the environment.

### Limitations of the Study

Although this study greatly accelerates the activation of PMS and reduces secondary pollution compared with some other systems, the amount of catalyst needed for the reaction is relatively high. Fe(II) is inevitably needed to activate PMS because MoO_2_ itself cannot activate PMS.

## Methods

All methods can be found in the accompanying Transparent Methods supplemental file.

## Supplemental Information

Document S1. Transparent Methods, Figures S1–S22, Scheme S1, and, Tables S1–S3

## References

[bib1] Al-Ghouti M.A., Khraisheh M.A.M., Allen S.J., Ahmad M.N. (2003). The removal of dyes from textile wastewater: a study of the physical characteristics and adsorption mechanisms of diatomaceous earth. J. Environ. Manage..

[bib2] Anipsitakis G.P., Dionysiou D.D. (2003). Degradation of organic contaminants in water with sulfate radicals generated by the conjunction of peroxymonosulfate with cobalt. Environ. Sci. Technol..

[bib3] Anipsitakis G.P., Dionysiou D.D. (2004). Radical generation by the interaction of transition metals with common oxidants. Environ. Sci. Technol..

[bib4] Barros D., Bouchet J., Raoult I., Mogne T., Martin J.M., Kasrai M., Yamada Y. (2003). Friction reduction by metal sulfides in boundary lubrication studied by XPS and XANES analyses. Wear.

[bib5] Brandt B.G. (1971). On the Crystal Structures of MoO_2_ and MoO_3_.2H_2_O: An Account of Computer Programming and Structure Refinement.

[bib6] Buck C., Skillen N., Robertson J., Robertson P.K.J. (2018). Photocatalytic OH radical formation and quantification over TiO_2_ P25: producing a robust and optimised screening method. Chin. Chem. Lett..

[bib7] Camacho-López M.A., Escobar-Alarcón L., Picquart M., Arroyo R., Córdoba G., Haro-Poniatowski E. (2011). Micro-Raman study of the m-MoO_2_ to α-MoO_3_ transformation induced by cw-laser irradiation. Opt. Mater..

[bib8] Chen C., Zuo W., Yang J., Cui H., Fu M. (2016). Yolk–shell structured CoFe_2_O_4_ microspheres as novel catalysts for peroxymonosulfate activation for efficient degradation of butyl paraben. RSC Adv..

[bib9] Chen J., Fang C., Xia W., Huang T., Huang C. (2018). Selective transformation of Î^2^-Lactam antibiotics by peroxymonosulfate: reaction kinetics and non-radical mechanism. Environ. Sci. Technol..

[bib10] Clarizia L., Russo D., Somma I.D., Marotta R., Andreozzi R. (2017). Homogeneous photo-Fenton processes at near neutral pH: a review. Appl. Catal. B Environ..

[bib11] Crini G. (2006). Non-conventional low-cost adsorbents for dye removal: a review. Bioresour.Technol..

[bib12] Dai D., Yang Z., Yao Y., Chen L., Jia G., Luo L. (2017). Highly efficient removal of organic contaminant based on peroxymonosulfate activation by iron phthalocyanine: mechanism and bicarbonate ion enhancement effect. Catal. Sci. Technol..

[bib13] Dan C., Ma X., Zhou J., Xi C., Qian G. (2014). Sulfate radical-induced degradation of Acid Orange 7 by a new magnetic composite catalyzed peroxymonosulfate oxidation process. J. Hazard. Mater..

[bib14] Dong C., Liu J., Xing M., Zhang J. (2018). Development of titanium oxide-based mesoporous materials in photocatalysis. Res. Chem. Intermediat..

[bib15] Du J., Bao J., Liu Y., Kim S.H., Dionysiou D.D. (2018). Facile preparation of porous Mn/Fe_3_O_4_ cubes as peroxymonosulfate activating catalyst for effective bisphenolA degradation. Chem. Eng. J..

[bib16] Du M., Qiu B., Zhu Q., Xing M., Zhang J. (2018). Cobalt phosphide nanocages encapsulated with graphene as ultralong cycle life anodes for reversible lithium storage. Res. Chem. Intermediat..

[bib17] Duan X., Su C., Miao J., Zhong Y., Shao Z., Wang S., Sun H. (2018). Insights into perovskite-catalyzed peroxymonosulfate activation: maneuverable cobalt sites for promoted evolution of sulfate radicals. Appl. Catal. B Environ..

[bib18] Fang G., Liu C., Wang Y., Dionysiou D.D., Zhou D. (2017). Photogeneration of reactive oxygen species from biochar suspension for diethyl phthalate degradation. Appl. Catal. B Environ..

[bib19] Furman O.S., Teel A.L., Watts R.J. (2010). Mechanism of base activation of persulfate. Environ. Sci. Technol..

[bib20] Ghanbari F., Moradi M. (2017). Application of peroxymonosulfate and its activation methods for degradation of environmental organic pollutants: Review. Chem. Eng. J..

[bib21] Guan Y., Ma J., Li X., Fang J., Chen L. (2011). Influence of pH on the formation of sulfate and hydroxyl radicals in the UV/peroxymonosulfate system. Environ. Sci. Technol..

[bib22] Hanawa T., Hiromoto S., Asami K. (2001). Characterization of the surface oxide film of a Co–Cr–Mo alloy after being located in quasi-biological environments using XPS. Appl. Surf. Sci..

[bib23] Harvey A.E., Smart J.A., Amis E.S. (1955). Simultaneous spectrophotometric determination of iron(II) and total iron with 1,10-phenanthroline. Anal. Chem..

[bib24] Hayon E., Treinin A., Wilf J. (1972). Electronic spectra, photochemistry, and autoxidation mechanism of the sulfite-bisulfite-pyrosulfite systems.SO_2_^-^, SO_3_^-^, SO_4_^-^, and SO_5_^-^ radicals. J. Am. Chem. Soc..

[bib25] Herrera L., Ruiz P., Aguillon J.C., Fehrmann A. (1989). A new spectrophotometric method for the determination of ferrous iron in the presence of ferric iron. J. Chem. Tech. Biotechnol..

[bib26] Hu B., Mai L., Wen C., Fan Y. (2009). From MoO_3_nanobelts to MoO_2_nanorods: structure transformation and electrical transport. ACS Nano.

[bib27] Hu P., Su H., Chen Z., Yu C., Li Q., Zhou B., Alvarez P.J.J., Long M. (2017). Selective degradation of organic pollutants using an efficient metal-free catalyst derived from carbonized polypyrrole via peroxymonosulfate activation. Environ. Sci. Technol..

[bib28] Huang G., Wang C., Yang C., Guo P., Yu H. (2017). Degradation of bisphenol A by peroxymonosulfatecatalytically activated with Mn_1.8_Fe_1.2_O_4_nanospheres: synergism between Mn and Fe. Environ. Sci. Technol..

[bib29] Ito T., Adachi Y., Yamanashi Y., Shimada Y. (2016). Long-term natural remediation process in textile dye-polluted river sediment driven by bacterial community changes. Water Res..

[bib30] Khan S., He X., Khan H.M., Boccelli D., Dionysiou D.D. (2016). Efficient degradation of lindane in aqueous solution by iron (II) and/or UV activated peroxymonosulfate. J. Photochem. Photobiol. A.

[bib31] Kusic H., Peternel I., Ukic S., Koprivanac N., Bolanca T., Papic S., Bozic A.L. (2011). Modeling of iron activated persulfate oxidation treating reactive azo dye in water matrix. Chem. Eng. J..

[bib32] Li H., Shan C., Pan B. (2018). Fe(III)-Doped g-C_3_N_4_ mediated peroxymonosulfate activation for selective degradation of phenolic compounds via high-valent iron-oxo species. Environ. Sci. Technol..

[bib33] Liang C., Su H. (2009). Identification of sulfate and hydroxyl radicals in thermally activated persulfate. Ind. Eng. Chem. Res..

[bib34] Ling S., Wang S., Peng Y. (2010). Oxidative degradation of dyes in water using Co^2+^/H_2_O_2_ and Co^2+^/peroxymonosulfate. J. Hazard. Mater..

[bib35] Liu J., Zhou J., Ding Z., Zhao Z., Xu X., Fang Z. (2017). Ultrasound irritation enhanced heterogeneous activation of peroxymonosulfate with Fe_3_O_4_ for degradation of azo dye. Ultrason. Sonochem..

[bib36] Muthuraman G., Teng T.T. (2009). Extraction of methyl red from industrial wastewater using xylene as an extractant. Prog. Nat. Sci..

[bib37] Pignatello J.J., Oliveros E., MacKay A. (2006). Advanced oxidation processes for organic contaminant destruction based on the fenton reaction and related chemistry. Crit. Rev. Environ. Sci. Technol..

[bib38] Pimentel M., Oturan N., Dezotti M., Oturan M.A. (2008). Phenol degradation by advanced electrochemical oxidation process electro-Fenton using a carbon felt cathode. Appl. Catal. B Environ..

[bib39] Rastogi A., Al-Abed S.R., Dionysiou D.D. (2009). Effect of inorganic, synthetic and naturally occurring chelating agents on Fe(II) mediated advanced oxidation of chlorophenols. Water Res..

[bib40] Rastogi A., Al-Abed S.R., Dionysiou D.D. (2009). Sulfate radical-based ferrous–peroxymonosulfate oxidative system for PCBs degradation in aqueous and sediment systems. Appl. Catal. B Environ..

[bib41] Sheng B., Yang F., Wang Y., Wang Z., Li Q., Guo Y., Lou X., Liu J. (2019). Pivotal roles of MoS_2_ in boosting catalytic degradation of aqueous organic pollutants by Fe(II)/PMS. Chem. Eng. J..

[bib42] Sun Y., Hu X., Luo W., Huang Y. (2011). Self-assembled hierarchical MoO_2_/graphenenanoarchitectures and their application as a high-performance anode material for lithium-ion batteries. ACS Nano.

[bib43] Tang R., Jiang C., Qian W., Jian J., Zhang X., Wang H., Yang H. (2015). Dielectric relaxation, resonance and scaling behaviors in Sr_3_Co_2_Fe_24_O_41_hexaferrite. Sci. Rep..

[bib44] Tao X., Ma W., Zhang T., Zhao J. (2001). Efficient photooxidative degradation of organic compounds in the presence of iron tetrasulfophthalocyanine under visible light irradiation. Angew.Chem. Int. Ed..

[bib45] Timmins G.S., Liu K.J., Bechara E.J., Kotake Y., Swartz H.M. (1999). Trapping of free radicals with direct in vivo EPR detection: a comparison of 5, 5-dimethyl-1-pyrroline-N-oxide and 5-diethoxyphosphoryl-5-methyl-1-pyrroline-N-oxide as spin traps for HO and SO_4_^⋅−^. Free Radic. Biol. Med..

[bib46] Tokarz-Sobieraj R., Gryboś R., Witko M. (2011). Electronic structure of MoO_2_. DFT periodic and cluster model studies. Appl. Catal. A.Gen..

[bib47] Ugo P., Moretto L.M., De Boni A., Scopece P., Mazzocchin G.A. (2002). Iron (II) and iron (III) determination by potentiometry and ion-exchange voltammetry at ionomer-coated electrodes. Anal. Chim. Acta.

[bib48] Wacławek S., Grübel K., Černík M. (2015). Simple spectrophotometric determination of monopersulfate. Spectrochim. Acta A.

[bib49] Wang S., Xu W., Wu J., Gong Q., Xie P. (2020). Improved sulfamethoxazole degradation by the addition of MoS_2_ into the Fe^2+^/peroxymonosulfate process. Sep. Purif. Technol..

[bib50] Xia X., Deng S., Xie D., Wang Y., Feng S., Wu J., Tu J. (2018). Boosting sodium ion storage by anchoring MoO_2_ on vertical graphene arrays. J. Mat. Chem. A.

[bib51] Xie X., Lin L., Liu R.Y., Jiang Y.F., Zhu Q., Xu A.W. (2015). The synergistic effect of metallic molybdenum dioxide nanoparticle decorated graphene as an active electrocatalyst for an enhanced hydrogen evolution reaction. J. Mater. Chem. A.

[bib52] Xing M., Xu W., Dong C., Bai Y., Zeng J., Zhou Y., Zhang J., Yin Y. (2018). Metal sulfides as excellent cocatalysts for H_2_O_2_ decomposition in advanced oxidation processes. Chem.

[bib53] Yang X., Cheng X., Elzatahry A.A., Chen J., Alghamdi A., Deng Y. (2019). Recyclable Fenton-like catalyst based on zeolite Y supported ultrafine, highly-dispersed Fe_2_O_3_ nanoparticles for removal of organics under mild conditions. Chin. Chem. Lett..

[bib60] Yi Q., Ji J., Shen B., Dong C., Liu J., Zhang J., Xing M. (2019). Singlet oxygen triggered by superoxide radicals in a molybdenum cocatalytic fenton reaction with enhanced REDOX activity in the environment.. Environ. Sci. Technol..

[bib54] Yi Q., Zhou Y., Xing M., Zhang J. (2015). Vacuum activation-induced Ti^3+^ and carbon codoped TiO_2_ with enhanced solar light photo-catalytic activity. Res. Chem. Intermediat..

[bib55] Yun E.T., Lee J.H., Kim J., Park H.D., Lee J. (2018). Identifying the nonradical mechanism in the peroxymonosulfate activation process: singlet oxygenation versus mediated electron transfer. Environ. Sci. Technol..

[bib56] Zamora P.L., Villamena F.A. (2012). Theoretical and experimental studies of the spin trapping of inorganic radicals by 5,5-dimethyl-1-pyrroline N-oxide (DMPO). 3. Sulfur dioxide, sulfite, and sulfate radical anions. J. Phys. Chem. A.

[bib57] Zhang T., Chen Y., Leiknes T. (2016). Oxidation of refractory benzothiazoles with PMS/CuFe_2_O_4_: kinetics and transformation intermediates. Environ. Sci. Technol..

[bib58] Zhang M., Chen M., Bi Y., Huang L., Zhou K., Zheng Z. (2019). A bimetallic Co_4_Mo_8_ cluster built from Mo_8_oxothiomolybdate capped by a Co_4_-thiacalix[4]arene unit: the observation of the Co–Mo synergistic effect for binder-free electrocatalysts. J. Mat. Chem. A.

[bib59] Zou J., Ma J., Chen L., Li X., Guan Y., Xie P., Pan C. (2013). Rapid acceleration of ferrous iron/peroxymonosulfate oxidation of organic pollutants by promoting Fe(III)/Fe(II) cycle with hydroxylamine. Environ. Sci. Technol..

