# Supplementary material for: Metallic Active Sites on MoO2(110) Surface to Catalyze Advanced Oxidation Processes for Efficient Pollutant Removal
Source: iScience. 2020 Jan 23;23(2):100861. doi: 10.1016/j.isci.2020.100861 (PMC7011042; doi:10.1016/j.isci.2020.100861)
Supplement: Document S1. Transparent Methods, Figures S1–S22, Scheme S1, and, Tables S1–S3 [file mmc1.pdf]

iScience, Volume *23*

Supplemental Information

Metallic Active Sites on MoO_2(110) Surface to Catalyze Advanced Oxidation Processes for Efficient Pollutant Removal

Jiahui Ji, Rashed M. Aleisa, Huan Duan, Jinlong Zhang, Yadong Yin, and Mingyang Xing

Supplemental Information

Metallic Active Sites on MoO_2 (110) Surface to Catalyze Advanced Oxidation Processes for Efficient Pollutant Removal

Jiahui Ji, Rashed M. Aleisa, Huan Duan, Jinlong Zhang, Yadong Yin and Mingyang Xing

SUPPLEMENTAL

Transparent Methods

Materials. All chemicals, including molybdenum dioxide (MoO_2, Shanghai Energy Chemical Co., Ltd., 99%), ferrous sulfate heptahydrate ($FeSO_4 \cdot 7H_2O$, Shanghai Aladdin Bio-Chem Technology Co., Ltd., 99.95%), potassium monopersulfate triple salt ($KHSO_5 \cdot 0.5KHSO_4 \cdot 0.5K_2SO_4$, PMS, Shanghai Macklin Biochemical Co., Ltd., 42% ~ 46% $KHSO_5$ basis), Lissamine rhodamine B (L-RhB, Shanghai Aladdin Bio-Chem Technology Co., Ltd.), phenol (Shanghai Aladdin Bio-Chem Technology Co., Ltd., ≥ 99%), methylene blue (MB, Shanghai Adamas Reagent Co., Ltd., RG, ≥ 98%), sulfadiazine (Shanghai Aladdin Bio-Chem Technology Co., Ltd., 98%), norfloxacin (Shanghai Aladdin Bio-Chem Technology Co., Ltd., 98%) 1,10-phenanthroline (Shanghai Lingfeng Chemical Reagent Co., Ltd., ≥ 99%), sodium hydroxide (NaOH, Shanghai Titan Scientific Co. Ltd., AR, ≥ 96.0%), sulfuric acid (H_2SO_4, Shanghai Titan Scientific Co. Ltd., CP, 95.0% ~ 98.0%), methanol (MeOH, Shanghai Aladdin Bio-Chem Technology Co., Ltd., AR, 99.5%), tert-butyl alcohol (TBA, Shanghai Aladdin Bio-Chem Technology Co., Ltd., GR, ≥ 99.5%), benzoic acid (Shanghai Lingfeng Chemical Reagent Co., Ltd., AR, ≥ 99.5%), 5,5-dimethyl-1-pyrroline N-oxide (DMPO, Shanghai Adamas Reagent Co., Ltd., RG, ≥ 98%) and potassium thiocyanate (KSCN, Shanghai Titan Scientific Co. Ltd., AR, ≥ 98.5%), were used without further purification. Deionized water (DI-water) was produced by OKP-S040 Standard ultrapure water system and used in all experiments.

Experimental Procedures. All experiments were performed in plastic cups with magnetic stirring to keep the solution homogeneous during the reaction. The predesigned initial pH of the L-RhB solution was adjusted first with NaOH, H_2SO_4 or buffer solutions. Then, fixed amounts of MoO_2 and $FeSO_4$•$7H_2O$ were added to 100 mL reaction solutions with the desired concentration of organic pollutants. Finally, quantitative PMS was added to initiate the oxidation. Samples were taken out at regular intervals, centrifuged and analyzed immediately. The stability of MoO_2 was also investigated. After being centrifuged, washed, dried and vacuum calcination/UV (365 nm)-activated, MoO_2 continued to participate in the next degradation reaction.

Radical quenching tests. Radical quenching tests were conducted to identify the dominant radicals in PMS/Fe(II)/MoO_2 system with methanol and TBA, which were added before the addition of PMS. The other procedures were the same as the experiments above. The radical species were further detected by electron paramagnetic resonance (EPR) technology, where 5,5-dimethyl-1-pyrroline (DMPO) was used as a spin-trapping reagent. The detailed parameters were as follows: a center field of 352.0 mT, a sweep width of 20.0 mT, a microwave frequency of 9.882 GHz, a microwave power of 6.402 mW, a temperature of 300.0 K, a receiver gain of 7.96×10^4, a modulation amplitude of 0.1 mT, and a sweep time of 41.94 s.

The variety of iron ions concentrations. The variety of Fe(II) or Fe(III) concentration was tracked during the reaction by complexing the samples with 1, 10-phenanthroline or potassium thiocyanate (KSCN), respectively. Fe(II) and Fe(III) can be complexed with 1,10-philoxene and KSCN for color development, respectively. Since the concentration of the complex is proportional to the absorbance, the corresponding absorbance of Fe(II)/Fe(III)-complex with the initial known concentration was measured. Thus, the content of Fe(II)/Fe(III) can be calculated from the ratio of the absorbance of the complex to the absorbance of the initial complex at different time periods: The L-RhB solution was replaced by deionized water while the other conditions and procedures remained unchanged; excessive 1,10-phenanthroline or potassium thiocyanate reagent was immediately added after sampling and centrifuging, and then analyzed.

The effect of dissolved Mo ions. Moreover, in order to investigate the effect of dissolved Mo ions for (i) the conversion of Fe(III)/Fe(II) and (ii) the degradation of L-RhB, the following experiments were also explored: MoO_2 was added first in (i) deionized water or (ii) L-RhB solution at fixed initial pH. Then, the solution was stirred for 30 min to dissolute Mo ions and centrifuged to remove solid MoO_2. Finally, $FeSO_4 \cdot 7H_2O$ and PMS was added to initiate the reaction. (i) Excessive 1,10-phenanthroline reagent was immediately added after sampling and centrifuging, and then analyzed; (ii) samples were taken out at regular intervals, centrifuged and analyzed immediately.

All the experiments in PMS/Fe(III)/MoO_2 system were the same as those in PMS/Fe(II)/MoO_2 system, just using Fe(III) to replace Fe(II).

Analytic Methods. The concentrations of L-RhB, MB, Fe(II) and Fe(III) were measured with a UV-Vis spectrophotometer (SHIMADZU UV-2450). The pH value and pH variation of the solution during the reaction were determined with a pH meter (INESA PHS-3C). The zeta potential of MoO_2 was measured for its isoelectric point (IEP) by a ZETASIZER instrument (Malvern ZEN3600). The morphology of MoO_2 was characterized by scanning electron microscope (SEM, JEOL JSM-6360 LV). X-ray diffraction (XRD) patterns were acquired in the range of 5-80° (2θ) by a RigakuD/MAX 2550 diffractometer, with the operation parameters of 40 kV and 100 mA and Cu Kα radiation (λ = 1.5406 Å). Raman spectroscopy was done using a Renishaw Invia spectrometer using a 532 nm Ar^+ laser at room temperature. X-ray photoelectron spectroscopy (XPS) of MoO_2 was conducted at a condition of Al Kα irradiation by THERMO ESCALAB 250 Xi. The total organic carbon (TOC) concentration of the filterable degradation agent was investigated using the SHIMADZU TOC-L CPN analyzer. The dissolved Mo ions in acidic conditions were detected by an inductively coupled plasma atomic emission spectrometer (ICP, NYSE: A 725). A PC fluorescence spectrophotometer (SHIMADZU RF-5301) was employed to obtain the photoluminescence (PL) spectroscopy of hydroxybenzoic acid to detect $^{\bullet}OH$. A high-performance liquid chromatography (HPLC, SHIMADZU LC-20A) were employed to explore the degradation of L-RhB, phenol, sulfadiazine and

norfloxacin. A gas chromatography-mass spectrometer (GC-MS, ThermoFisher Trace ISQLT) with HP-5ms column (30 m * 250 um * 0.25 um) was employed to explore the intermediates in the degradation process of phenol. The heating program was maintained at 40 °C for 3 min, heated to 300 °C at 5 °C/min, and held for 10 min. The inlet temperature was 300 °C, the transfer line temperature was 300 °C, and the column flow rate was 1.2 mL/min. Mass spectrometry conditions: EI ionization source (70 eV, full scan).

DFT calculation. Computational details. Density functional theory (DFT) calculations were carried out using the all-electron code Fritz-Haber Institute ab initio molecules simulations package (FHI-aims).(Blum et al., 2009) Interactions between atomic core shells and the valence electrons were described using the projector-augmented wave (PAW) method, the Perdew-Burke-Ernzerh (PBE) of gradient-corrected functional was used to treat the exchange and correlation. (Perdew, 1996, Kresse, 1999) The default "tight" species were chosen in this work. Considering the weak non-covalent van der Waals attraction, all calculations were performed by the scheme of Tkatchenko and Scheffler. (Tkatchenko and Scheffler, 2009) Gaussian smearing was used with a width of 0.1 eV to determine the partial occupancies. The convergence threshold was set to be 10^{-5} eV in energy and 10^{-3} eV·Å^{-1} in force. A periodic (110) slab model of MoO_2 was built up to simulate the activation process of peroxymonosulfate (PMS) molecules, which preferred to adsorb on the (110) surfaces, contributed to the electron transfer and the generation of hydroxyl radicals. Besides, the catalytic effect of Fe(II) ions was also taken into account for low surface coverages of PMS molecules. To minimize the

interaction of (110) surfaces in different supercells along c direction, a 20 Å vacuum layer between them was taken. All atoms were allowed to relax expected the bottom three-layer atoms to fix at the bulk parameters. Brillouin zone was sampled using a 3×3×1 Monkhorst Pack k-point mesh during geometry optimization and properties calculation for the (110) surface of MoO_2. (Monkhorst, 1976)

Adsorption energy and charge transfer. To reveal the activation process of PMS molecule on the (110) surface of MoO_2, the interaction between them could be evaluated by the adsorption energies, which was defined as $\Delta E_{ads}=E_{MoO_2+PMS}-E_{MoO_2}-E_{PMS}$, where E_{MoO_2+PMS} was the total energy for the PMS adsorbed on the surface, E_{MoO_2} was the total energy for the MoO_2 substrate without adsorption, and E_{PMS} was the total energy of a separated molecule as determined from DFT calculations. The Mulliken charge analysis was used to quantitatively estimate the amount of charge transfer between the adsorbed molecule and MoO_2 substrate, which contributed to reveal the activation mechanism. (Tang et al., 2009)

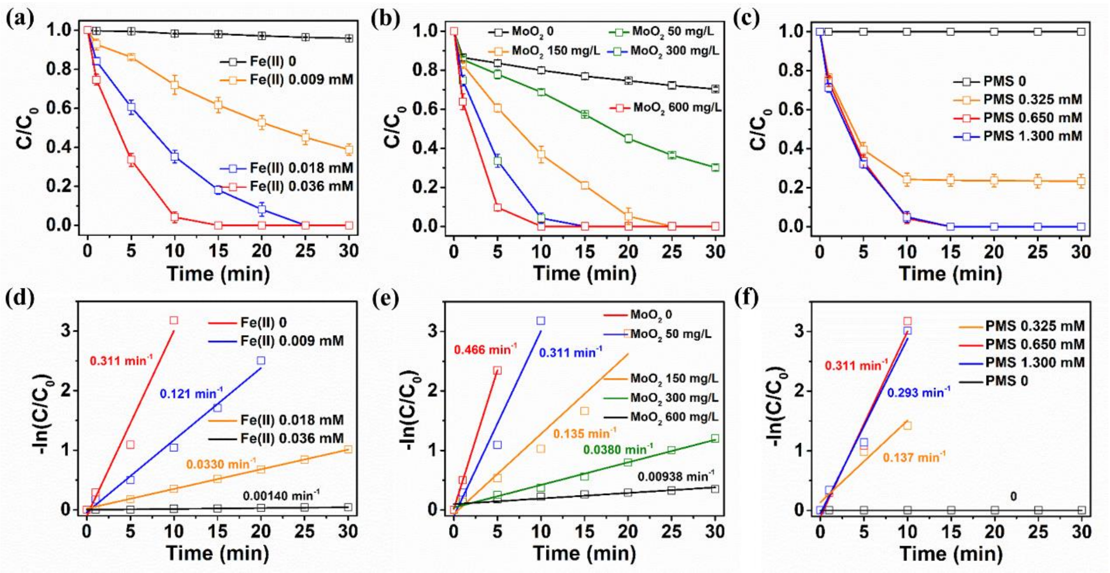

Figure S1. Effect of (a) Fe(II) concentration, (b) MoO_2 concentration, (c) PMS concentration on L-RhB oxidation in PMS/Fe(II)/MoO_2 system; Pseudo-first-order kinetics of effect of (d) Fe(II), (e) MoO_2 and (f) PMS concentration. General conditions: $[PMS]_0 = 0.650$ mM, $[Fe(II)]_0 = 0.036$ mM, $[MoO_2]_0 = 300$ mg/L, initial pH = 3.0, $[L\text{-}RhB]_0 = 20$ mg/L. Error bars represent the standard deviation from at least duplicate experiments. Related to Figure **1**.

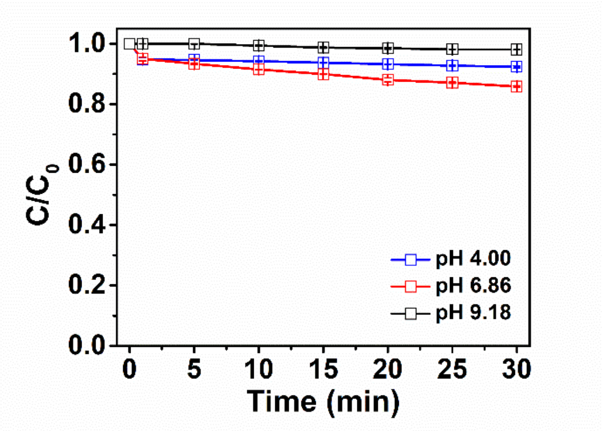

Figure S2. The investigation of the influence of solution pH with potassium hydrogen phthalate ($C_8H_5KO_4$, pH 4.00), mixed phosphate (pH 6.86) and borax ($Na_2B_4O_7 \cdot 10H_2O$, pH 9.18) buffer solutions, respectively. General conditions: $[PMS]_0$ = 0.650 mM, $[Fe(II)]_0$ = 0.036 mM, $[MoO_2]_0$ = 300 mg/L, initial pH = 3.0, $[L\text{-}RhB]_0$ = 20 mg/L. Error bars represent the standard deviation from at least duplicate experiments. Related to Figure **1**.

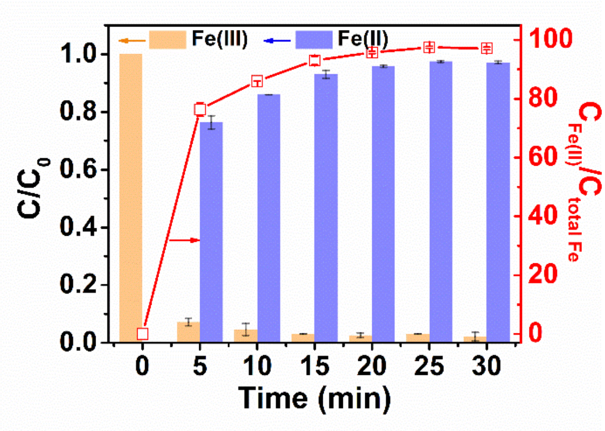

Figure S3. The variation of Fe(II) and Fe(III) concentrations in Fe(III)/MoO_2 system. General conditions: $[Fe(III)]_0$ = 0.035 mM (total Fe), $[MoO_2]_0$ = 300 mg/L, initial pH = 3.0. Error bars represent the standard deviation from at least duplicate experiments. Related to Figure **2**.

The reduction potential of Fe(III)/Fe(II) (0.77 V) is higher than that of MoO_4^{2-}/MoO_2 (0.65 V). As shown in Fig. S5, when MoO_2 was added to the Fe(III)-containing solution, Fe(III) was immediately reduced to Fe(II), which was almost completely reduced within 15 min. Thus, it can be concluded that MoO_2 itself can reduce Fe(III) to Fe(II), which solves the problem that iron ions are difficult to circulate in PMS/Fe(II) system.

Catalyst	Catalyst dosage	PMS concen.	Organic pollutant	Removal efficiency	Ref.
MoO_2/Fe(II) (this work)	0.30 g·L^{-1} /0.036 mM	0.65 mM	L-RhB; 20 mg·L^{-1}	96% in 10 min	-
HA/Fe(II)	0.4 mM /10.8 μM	0.32 mM	BA; 40 μM	94% in 15 min	(Zou et al., 2013)
Fe_3O_4@C/Co	0.20 g·L^{-1}	0.1 g·L^{-1}	AO II; 20 mg·L^{-1}	40 min	(Xu et al., 2015)
Fe_3O_4@MnO_2 BBHs	0.30 g·L^{-1}	20 mM	MB; 20 mg·L^{-1}	30 min	(Zhang et al., 2016)
$Co_3[Fe(CN)_6]_2$	50 mg·L^{-1}	50 mg·L^{-1}	RhB; 10 mg·L^{-1}	20 min	(Lin et al., 2016)
CNF3	0.10 g·L^{-1}	1.0 mM	4-CP; 0.10 mM	20 min	(Li et al., 2018a)
FeCo-LDH	0.20 g·L^{-1}	0.15 g·L^{-1}	RhB; 20 mg·L^{-1}	10 min	(Gong et al., 2017)
Fe_3O_4/Mn_3O_4/rGO	0.10 g·L^{-1}	0.30 g·L^{-1}	MB; 50 mg·L^{-1}	93.5% in 30 min	(Yang et al., 2015)
M@N-C (M=Fe, Co)	20 mg·L^{-1}	0.65 mM	Orange II; 20 mg·L^{-1}	90 min	(Yao et al., 2016)
$CoFe_2O_4$	0.40 g·L^{-1}	0.8 mM	ATZ; 10 mg·L^{-1}	30 min	(Li et al., 2018b)
Fe_3O_4@C/$MnCo_2O_4$	0.15 g·L^{-1}	0.06 g·L^{-1}	AO II; 20 mg·L^{-1}	99% in 15 min	(Lu et al., 2017)

Table S1. The catalytic performance of PMS/Fe(II)/MoO_2 system compared with other reported catalysts. Related to Figure **1**.

**Valence state**	**IV**	**V+VI**
Mo (before reaction)	26.2%	73.8%
Mo (after reaction)	25.7%	74.3%

Table S2. The variety of Mo valence distribution percentage before and after reaction. Related to Figure **3**.

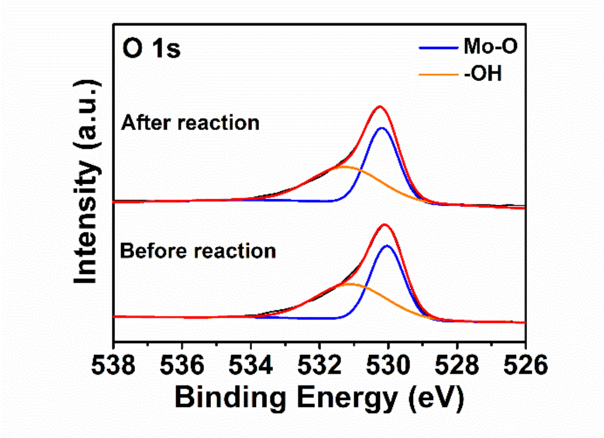

Figure S4. O1s spectra of MoO_2 before and after reaction in PMS/Fe(II)/MoO_2 system. Related to Figure **3**.

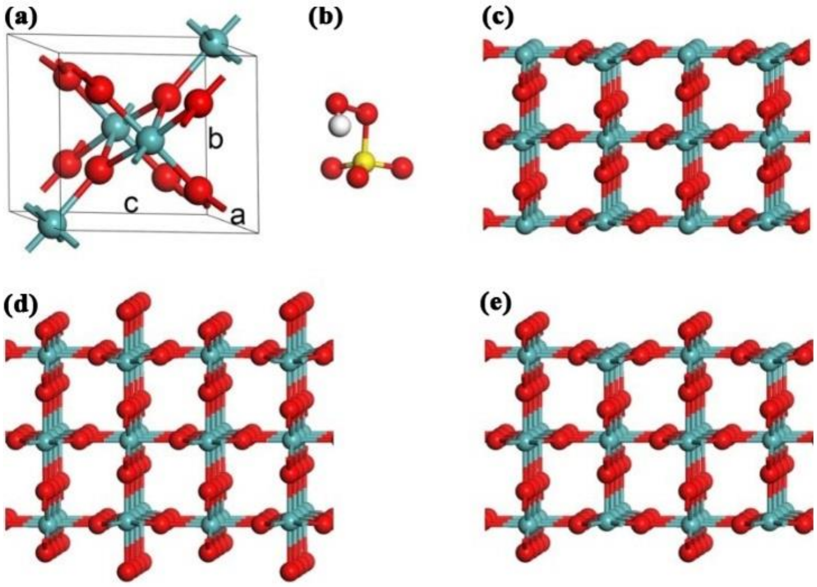

Figure S5. Optimized structure of MoO_2, PMS and three ideal terminations of (110) surface: (a) unite cell, (b) HSO_5^-, (c) the termination with exposed MoO active centers, (d) the termination with exposed O active centers, (e) the termination with exposed O’ active centers. The yellow, red, olive, and white atoms are S, O, Mo, and H atoms, respectively. Related to Figure **4**.

Types	ΔE_{ads} (eV)	Δq (e)	$l_{O\text{–}O}$ (Å)
Free HSO_5^- molecule	/	/	1.36
HSO_5^- on (110)	-2.06	0.62	1.40
HSO_5^- on Fe(II)-(110)	-3.17	0.75	1.48
SO_4^{2-}+$HO^{\cdot}$ on (110)	-1.68	0.69	/
SO_4^{2-}+$HO^{\cdot}$ on Fe(II)-(110)	-2.54	0.83	/

Table S3. The adsorption energy (ΔE_{ads}), the electron transfer between the molecule and MoO_2 (Δq), and the bond length ($l_{O\text{-}O}$) of $[SO_4\text{-}OH]^-$ in the different adsorption configurations. Related to Figure **4**.

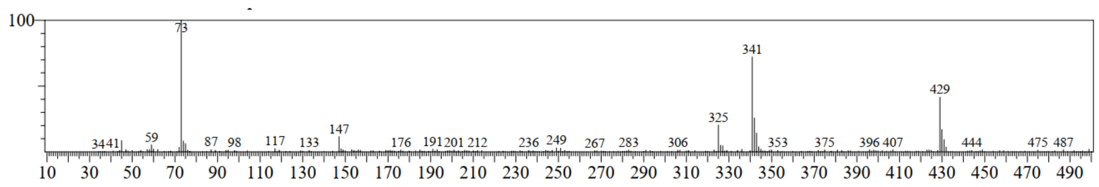

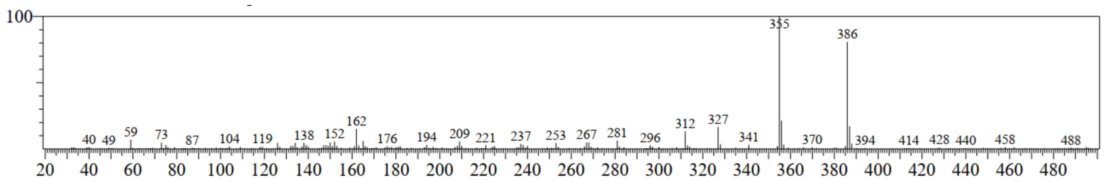

Figure S6. Mass spectrometry of phenol detected by GC-MS. Conditions: $[PMS]_0$ = 0.650 mM, $[Fe(II)]_0$ = 0.036 mM, $[MoO_2]_0$ = 300 mg/L, initial pH = 3.0, $[phenol]_0$ = 20 mg/L. Related to Figure **5**.

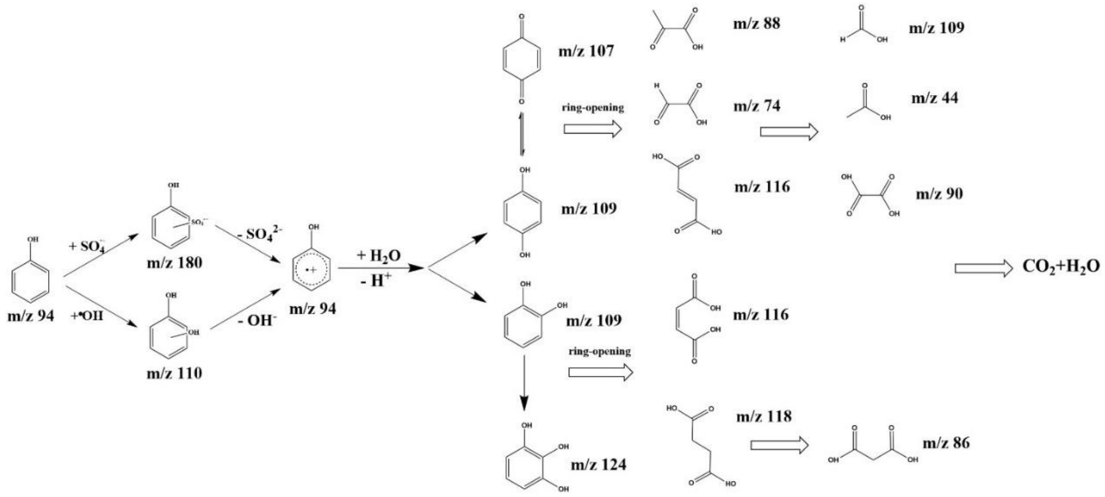

Scheme S1. Reaction pathway of phenol mineralization in the PMS/Fe(II)/MoO_2 system. Related to Figure **5**.

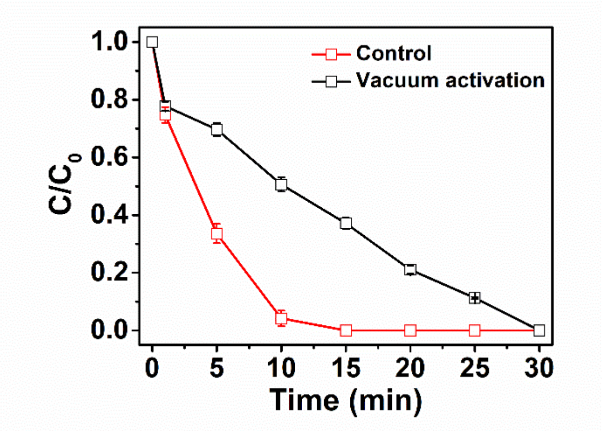

Figure S7. Recycling of MoO_2 after vacuum activation for PMS/Fe(II)/MoO_2 system. General conditions: $[PMS]_0$ = 0.650 mM, $[Fe(II)]_0$ = 0.036 mM, $[MoO_2]_0$ = 300 mg/L, initial pH = 3.0, $[L\text{-}RhB]_0$ = 20 mg/L. Error bars represent the standard deviation from at least duplicate experiments. Related to Figure **5**.

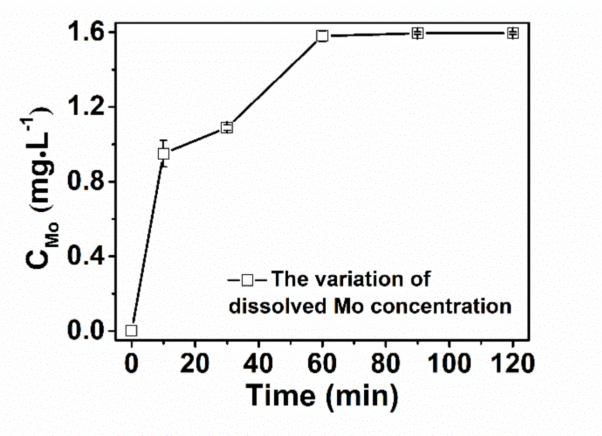

Figure S8. Dissolution of Mo ions in PMS/Fe(II)/ MoO_2 system. General conditions: $[PMS]_0$ = 0.650 mM, $[Fe(II)]_0$ = 0.036 mM, $[MoO_2]_0$ = 300 mg/L, initial pH = 3.0. Error bars represent the standard deviation from at least duplicate experiments. Related to Figure **5**.

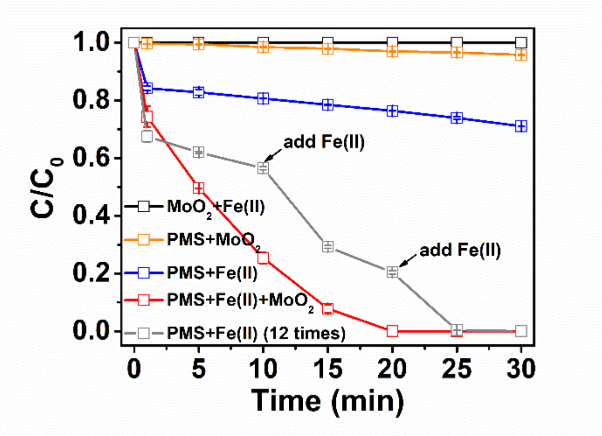

Figure S9. Scale up tests (1 L system). General conditions: $[PMS]_0$ = 0.650 mM, $[Fe(II)]_0$ = 0.036 mM, $[MoO_2]_0$ = 300 mg/L, initial pH = 3.0, $[L\text{-}RhB]_0$ = 20 mg/L. Error bars represent the standard deviation from at least duplicate experiments. Related to Figure **5**.

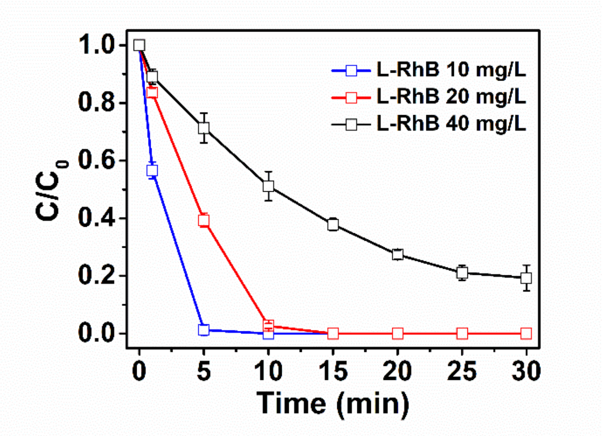

Figure S10. Degradation of different L-RhB concentration in PMS/Fe(III)/MoO_2 system. Conditions: $[PMS]_0 = 0.650$ mM, $[Fe(III)]_0 = 0.035$ mM, $[MoO_2]_0 = 300$ mg/L, initial pH = 3.0. Error bars represent the standard deviation from at least duplicate experiments. Related to Figure **5**.

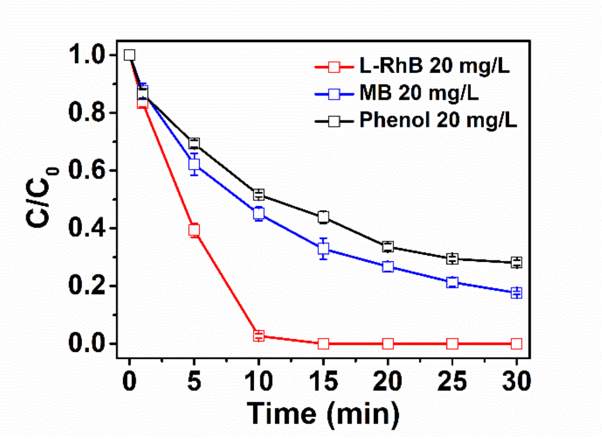

Figure S11. Degradation of different aromatic organic compounds in PMS/Fe(III)/MoO_2 system. Conditions: $[PMS]_0 = 0.650$ mM, $[Fe(III)]_0 = 0.035$ mM, $[MoO_2]_0 = 300$ mg/L, initial pH = 3.0, [aromatic organic compound]$_0$ = 20 mg/L. Error bars represent the standard deviation from at least duplicate experiments. Related to Figure **5**.

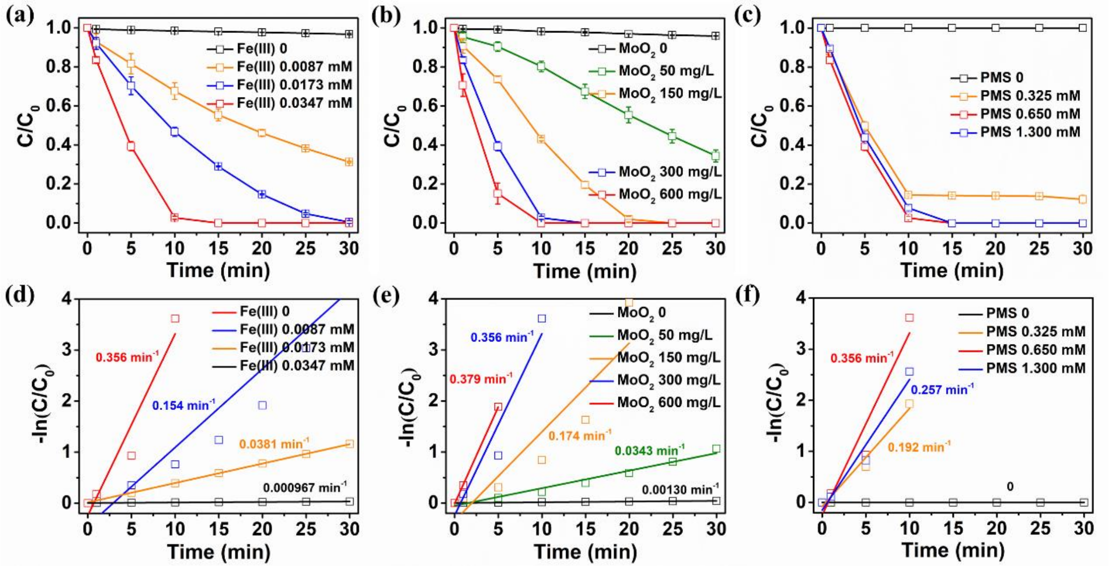

Figure S12. Effect of (a) Fe(III) concentration, (b) MoO_2 concentration, (c) PMS concentration on L-RhB degradation in PMS/Fe(III)/MoO_2 system; Pseudo-first-order kinetics of effect of (a) Fe(III), (b) MoO_2 and (c) PMS concentration in PMS/Fe(II)/MoO_2 system. Conditions: $[PMS]_0$ = 0.650 mM, $[Fe(III)]_0$ = 0.035 mM, $[MoO_2]_0$ = 300 mg/L, initial pH = 3.0, $[L\text{-}RhB]_0$ = 20 mg/L. Error bars represent the standard deviation from at least duplicate experiments. Related to Figure **5**.

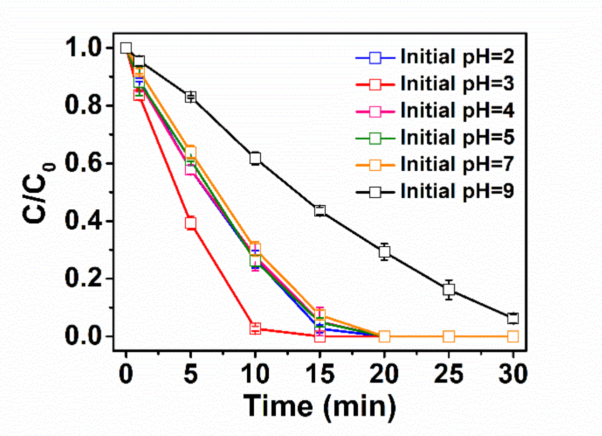

Figure S13. Effect of initial pH in PMS/Fe(III)/MoO_2 system. Conditions: $[PMS]_0$ = 0.650 mM, $[Fe(III)]_0$ = 0.035 mM, $[MoO_2]_0$ = 300 mg/L, initial pH = 3.0, $[L\text{-}RhB]_0$ = 20 mg/L. Error bars represent the standard deviation from at least duplicate experiments. Related to Figure **5**.

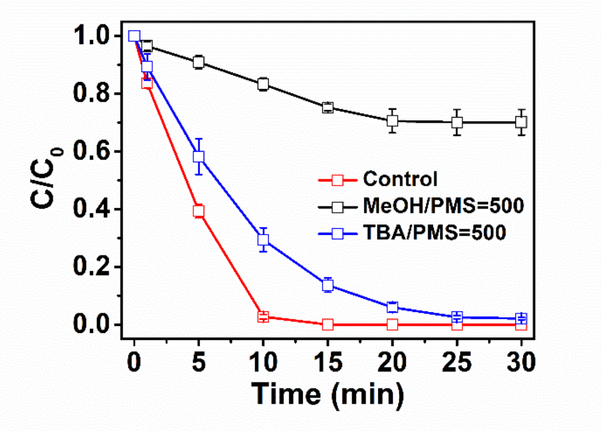

Figure S14. Inhibition effect of radical scavengers on L-RhB degradation in PMS/Fe(III)/MoO_2 system. Conditions: $[PMS]_0$ = 0.650 mM, $[Fe(III)]_0$ = 0.035 mM, $[MoO_2]_0$ = 300 mg/L, initial pH = 3.0, $[L\text{-}RhB]_0$ = 20 mg/L. Error bars represent the standard deviation from at least duplicate experiments. Related to Figure **5**.

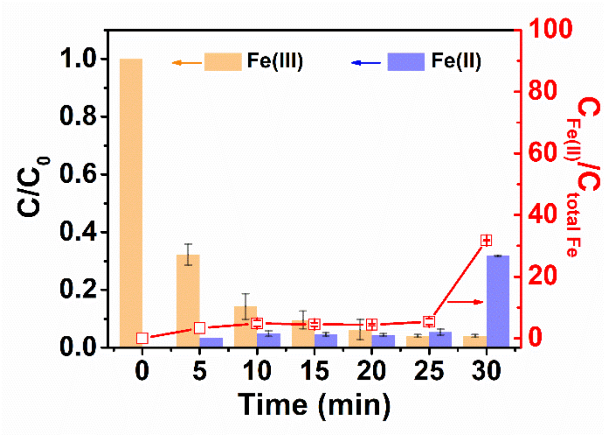

Figure S15. The variation of Fe(II) and Fe(III) concentrations in PMS/Fe(III)/MoO_2 system. General conditions: $[PMS]_0$ = 0.650 mM, $[Fe(III)]_0$ = 0.035 mM (total Fe), $[MoO_2]_0$ = 300 mg/L, initial pH = 3.0. Error bars represent the standard deviation from at least duplicate experiments. Related to Figure **5**.

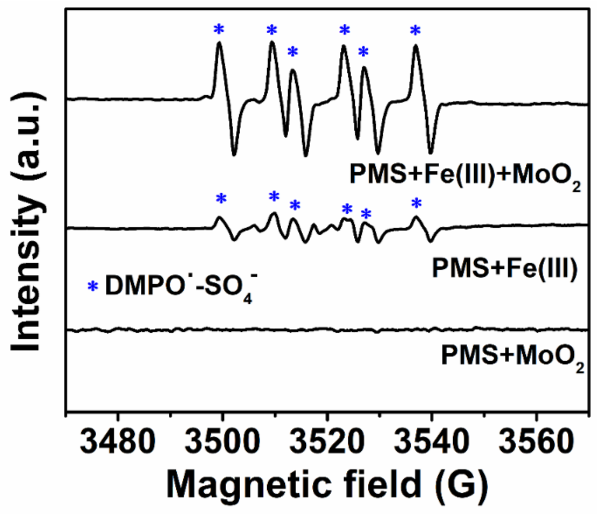

Figure S16. EPR spectra obtained from (i) PMS/MoO_2 system, (ii) PMS/Fe(III) system, and (iii) PMS/Fe(III)MoO_2 system with the existence of DMPO (* represents $SO_4^{\cdot -}$ adduct). Conditions: $[PMS]_0 = 0.650$ mM, $[Fe(III)]_0 = 0.035$ mM, $[MoO_2]_0 = 300$ mg/L, initial pH = 3.0. Related to Figure **5**.

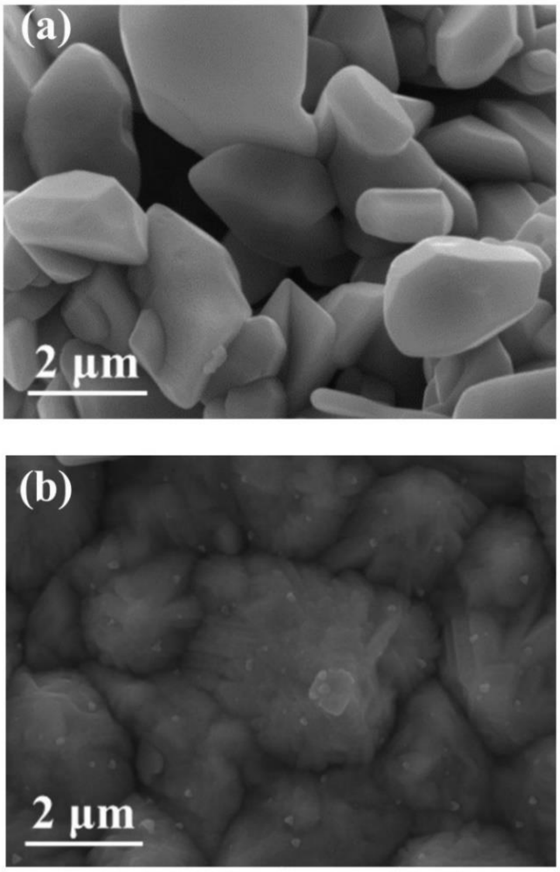

Figure S17. SEM images of MoO_2 (a) before and (b) after reaction in PMS/Fe(III)/MoO_2 system. Related to Figure **5**.

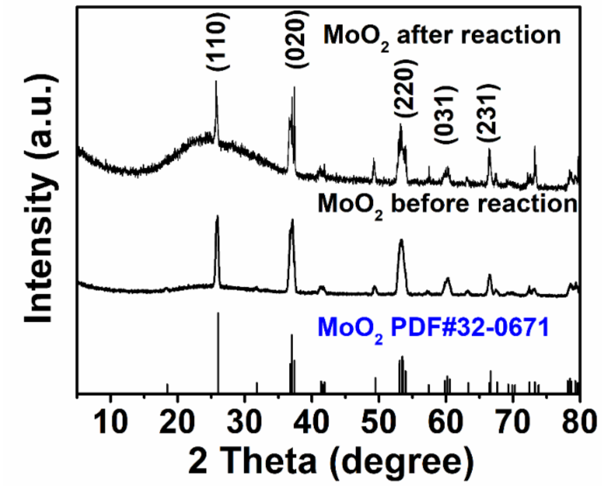

Figure S18. XRD patterns of MoO_2 before and after reaction in PMS/Fe(III)/MoO_2 system. Related to Figure **5**.

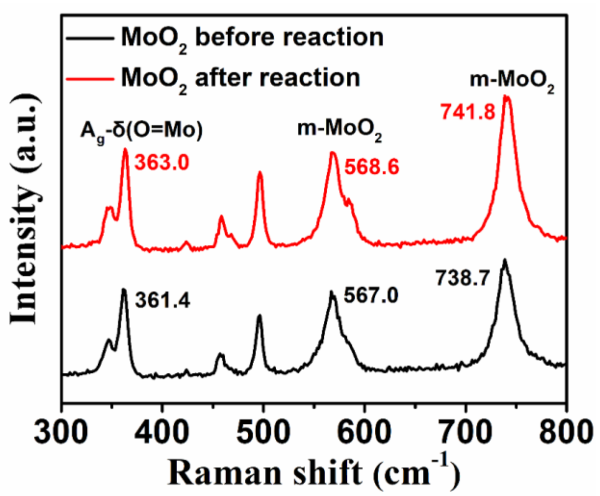

Figure S19. Raman spectra of MoO_2 before and after reaction in PMS/Fe(III)/MoO_2 system. Related to Figure **5**.

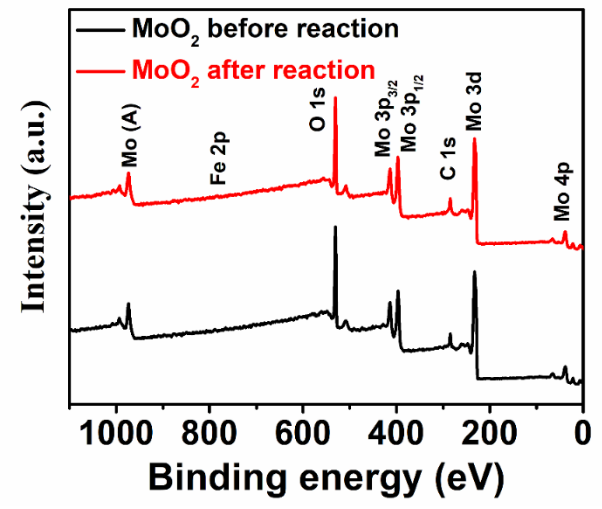

Figure S20. XPS survey spectra of MoO_2 before and after reaction in PMS/Fe(III)/MoO_2 system. Related to Figure **5**.

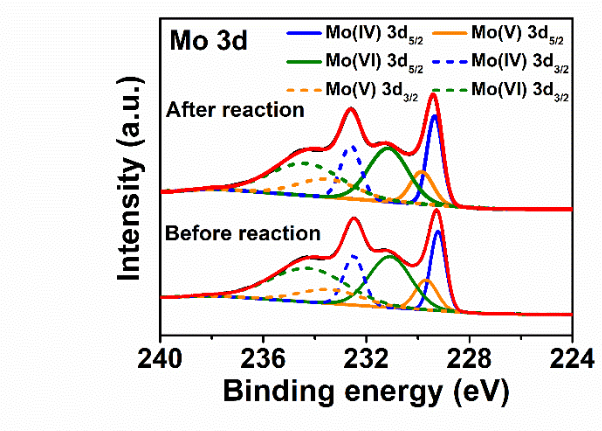

Figure S21. Mo3d spectra of MoO_2 before and after reaction in PMS/Fe(III)/MoO_2 system. Related to Figure **5**.

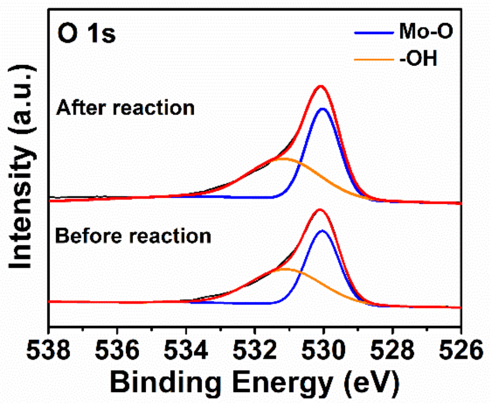

Figure S22. O1s spectra of MoO_2 before and after reaction in PMS/Fe(III)/MoO_2 system. Related to Figure **5**.

REFERENCES

Blum, V., Gehrke, R., Hanke, F., Havu, P., Havu, V., Ren, X., Reuter, K., and Scheffler, M. (2009). Ab initio molecular simulations with numeric atom-centered orbitals. Computer Phys. Commun. 180, 2175-2196.

Gong, C., Chen, F., Yang, Q., Luo, K., Yao, F., Wang, S., Wang, X., Wu, J., Li, X., Wang, D. and Zeng, G. (2017). Heterogeneous activation of peroxymonosulfate by Fe-Co layered doubled hydroxide for efficient catalytic degradation of Rhoadmine B. Chem. Eng. J. 321, 222-232.

Kresse, G., and Joubert, D. (1999). From ultrasoft pseudopotentials to the projector augmented-wave method. Phys. Rev. B 59, 1758-1775.

Li, H., Shan, C., and Pan, B. (2018a). Fe(III)-Doped g-C_3N_4 Mediated Peroxymonosulfate Activation for Selective Degradation of Phenolic Compounds via High-Valent Iron-Oxo Species. Environ. Sci. Technol. 52, 2197-2205.

Li, J., Xu, M., Yao, G. and Lai, B. (2018b). Enhancement of the degradation of atrazine through $CoFe_2O_4$ activated peroxymonosulfate (PMS) process: Kinetic, degradation intermediates, and toxicity evaluation. Chem. Eng. J. 348, 1012-1024.

Lin, K. Y. A., Chen, B. J., and Chen, C. K. (2016). Evaluating Prussian blue analogues $MII_3[MIII(CN)_6]_2$ (MII = Co, Cu, Fe, Mn, Ni; MIII = Co, Fe) as activators for peroxymonosulfate in water. RSC Adv. 6, 92923-92933.

Lu, J., Liu, Q., Xiong, Z., Xu, Z., Cai, Y., and Wang, Q. (2017). Activation of peroxymonosulfate with magnetic and recyclable Fe_3O_4@C/$MnCo_2O_4$ nanocomposites for the decolorization of Acid Orange II. J. Chem. Technol. Biot. 92, 1601-1612.

Monkhorst, H. J., and Pack, J. D. (1976). Special points for Brillouin-zone integrations. Phys. Rev. B 13, 5188-5192.

Perdew, J. P., Burke, K., and Ernzerhof, M. (1996). Generalized gradient approximation made simple. . Phys. Rev. Lett. 77, 3865-3868.

Perdew, J. P., Burke, K., and Ernzerhof, M. (2009). A grid-based Bader analysis algorithm without lattice bias. J. Phys. Condens. Matt. 21, 084204.

Tkatchenko, A. and Scheffler, M. (2009). Accurate molecular van der Waals interactions from ground-state electron density and free-atom reference data. Phys. Rev. Lett. 102, 073005.

Xu, Z., Lu, J., Liu, Q., Duan, L., Xu, A., Wang, Q., and Li, Y. (2015). Decolorization of Acid Orange II dye by peroxymonosulfate activated with magnetic Fe_3O_4@C/Co nanocomposites. RSC Adv. 5, 76862-76874.

Yang, B., Tian, Z., Wang, B., Sun, Z., Zhang, L., Guo, Y., Li, H. and Yan, S. (2015). Facile synthesis of Fe_3O_4/hierarchical-Mn_3O_4/graphene oxide as a synergistic catalyst for activation of peroxymonosulfate for degradation of organic pollutants. RSC Adv. 5, 20674-20683.

Yao, Y., Chen, H., Lian, C., Wei, F., Zhang, D., Wu, G., Chen, B. and Wang, S. (2016). Fe, Co, Ni nanocrystals encapsulated in nitrogen-doped carbon nanotubes as Fenton-like catalysts for organic pollutant removal. J. Hazard. Mater. 314, 129-139.

Zhang, S., Fan, Q., Gao, H., Huang, Y., Liu, X., Li, J., Xu, X. and Wang, X. (2016). Formation of Fe_3O_4@MnO_2 ball-in-ball hollow spheres as a high performance catalyst with enhanced catalytic performances. J. Mater. Chem. A 4, 1414-1422.

Zou, J., Ma, J., Chen, L., Li, X., Guan, Y., Xie, P. and Pan, C. (2013). Rapid acceleration of ferrous iron/peroxymonosulfate oxidation of organic pollutants by promoting Fe(III)/Fe(II) cycle with hydroxylamine. Environ. Sci. Technol. 47, 11685-91.
